# Companion animals and child development outcomes: longitudinal and cross-sectional analysis of a UK birth cohort study

**DOI:** 10.1186/s12887-024-05049-7

**Published:** 2024-09-13

**Authors:** Rebecca Purewal, Robert Christley, Katarzyna Kordas, Carol Joinson, Kerstin Meints, Nancy Gee, Carri Westgarth

**Affiliations:** 1 Department of Livestock and One Health, School of Veterinary Science, Institute of Infection, Veterinary and Ecological Sciences, Faculty of Health and Life Sciences, University of Liverpool, Leahurst Campus, Neston, CH64 7TE Cheshire UK; 2 Department of Epidemiology and Environmental Health, University at Buffalo, 270 Farber Hall, Buffalo, NY 14214 USA; 3 Population Health Sciences, Bristol Medical School, University of Bristol, 1-5 Whiteladies Road, Clifton, BS8 1NU Bristol UK; 4 School of Psychology, University of Lincoln, Brayford Wharf East, Lincoln, LN5 7AY Lincolnshire UK; 5 Center for Human-Animal Interaction, School of Medicine, Virginia Commonwealth University, Richmond, VA 23298-0710 USA

**Keywords:** ALSPAC, Animals, Child development, Dogs, Longitudinal studies, Ownership

## Abstract

**Background:**

Research into the impact of social relationships on childhood and adolescent health and wellbeing has been largely limited to children’s relationships with other humans, while studies into the impact of pet ownership are sparse and have generally not adjusted for potential confounders. This study aimed to investigate the association between pet ownership and a range of developmental outcomes in childhood and adolescence.

**Methods:**

Data were self-reports and direct assessments of approx. 14,000 children from the Avon Longitudinal Study of Parents and Children (ALSPAC). Multivariable regression models adjusting for confounding factors examined associations between developmental outcome measures (emotional health, behavioural development, cognitive development, language development, educational attainment) and concurrent pet ownership, including species, and also longitudinal pet ownership history and pet-interaction where possible. Analyses model numbers using multiple imputation varied from *n* = 393–8963.

**Results:**

In cross-sectional analyses, owning a dog (b = 0.24, [0.06–0.41], *p* = .004) and owning other/miscellaneous pets (b = 0.18, [0.03–0.33], *p* = .021) at age 3 were associated with higher prosocial behaviour score. Owning a pet was associated with a higher non-verbal communication score at age 2 (cross-sectional, b = 0.18, [0.04–0.32], *p* = .014), and a higher language development score at age 5 (cross-sectional, b = 1.01, [0.18–1.83], *p* = .017). However, pet ownership was associated with lower educational attainment across a number of academic subjects and timepoints, in both cross-sectional and longitudinal analyses. It was also cross-sectionally linked to hyperactivity at age 3 and conduct problems at age 3 and 11. Furthermore, at age 8, cross-sectional analysis showed that children who owned any pets (OR [95% CI]: 0.85 [0.73–0.98], p= ·026) or cats (0.83, [0.73–0.95], p= ·006) had lower odds of high self-esteem (scholastic competence).

**Conclusions:**

Using a large, well-designed longitudinal study and adjusting for key confounders, we found little evidence of cross-sectional or longitudinal associations between pet ownership and emotional health or cognitive outcomes in children. There may, however, be some cross-sectional and longitudinal association with poorer educational attainment and a positive impact on social interactions as seen through associations with enhanced language development and prosocial behaviour. This study demonstrates the importance of adjustment for confounding variables and suggests that, contrary to popular belief, positive impacts of pet ownership on childhood development may be mainly limited to social behaviour and language development.

**Supplementary Information:**

The online version contains supplementary material available at 10.1186/s12887-024-05049-7.

## Background

Childhood and adolescence are crucial life phases for contribution to the quality of health, emotional well-being, learning and behaviour across the life span. According to relationship psychology, social interaction theories [1, 2] and attachment theory [3–5], social relationships contribute fundamentally to child and adolescent development, yet studies have been largely limited to children’s relationships with other humans. In western cultures, pet ownership is common; 70% of U.S. households [6] and 59% of British households [7] include at least one companion animal. There is increasing research interest in the potential benefits of companion animals on human health [8–10], but the relationship between childhood animals and developmental outcomes is unclear [11].

### Emotional and behavioural development

Emotional and mental health difficulties, such as depression, separation and social anxiety disorder, are relatively common in Children and Young People and increasing. In 2017 the estimated prevalence in 5-19-year-olds in the UK was 8.1% [12], but a follow-up in 2022 suggests that 18% of 7–16 year olds had a ‘probable’ and 11% a ‘possible’ mental disorder [13]. The percentage of behavioural disorders (e.g., hyperactivity, conduct disorders) is slightly lower, for example in 2017 it was reported among 5% of UK 5–19 year olds [12]. Emotional difficulties that arise in childhood can persist into young adulthood [14, 15]. Due to increasing pressures on mental and behavioural health spending, and the difficulty in accessing specialist interventions, prevention and early intervention may be more cost-effective [16].

Interaction with animals can mediate human physiological responses to stressors and anxiety, and may improve mental, social, and physical health [17–19]. Interaction with animals can also affect the endocrine system. Oxytocin levels have been found to increase in the presence of a pet [19] and in turn can stimulate social interaction, increase social skills, increase positive self-perception, and decrease depressive symptoms [20, 21]. Oxytocin also has an anxiolytic effect for social anxiety [22] and social fear [23]. Interacting with dogs and cats has also been shown to lower the stress hormone, cortisol [17, 24].

Companion animals can act as social facilitators for interpersonal human social interactions (social-catalyst effect) which in turn may reduce loneliness, social anxiety and depression, and increase self-esteem [25]. Pets are also perceived to provide unconditional positive regard, approval, and acceptance without judgement, for example when these feelings or affirmations are lacking from caregivers or peers [26, 27]. Gaining this type of emotional support is essential for the healthy psychological development in childhood and adolescence, especially during periods of growth, developmental changes, and challenging social situations.

Animals have been involved with children in psychotherapy [28] and animal-assisted therapy [29], including in the classroom [30], with some evidence of positive outcomes. For example, significant effects on lowering of cortisol after 4 weeks of dog-assisted interventions in school children with and without special educational needs have been found [17]. However, involving animals in ‘therapy’ in settings such as schools is not completely free of criticism for example for welfare reasons [31], and the extent to which these findings might apply instead to the presence of companion animals in the home is unclear. It is also unknown which species/types may be most beneficial for certain age groups, and whether effects vary by developmental outcome. One recent study found that dog-owning adolescents showed significantly less psychological symptoms (sadness, depression, fear, and nervousness) than adolescents who reported having a cat, a dog and a cat, or having other pets [32].

### Cognitive and language development and educational attainment

Early enhancement of cognitive ability (executive function, general intelligence, and language) sets a child on a trajectory for success in later life. For example, executive functions (e.g., working memory, inhibitory control, cognitive flexibility, planning, reasoning, problem-solving) are predictive of achievement throughout life, often more so than IQ or socio-economic status [33]. Evidence suggests that early honing of executive functionss can reduce later incidence of school failure, aggression, and antisocial behaviour [34].

It is noteworthy that executive function and emotion regulation are interlinked and influence each other with inhibitory control and self-regulation overlapping to some extent [35]. Self-regulation may also impact key social and emotional skills that affect school readiness and later school success [36]. Various studies have demonstrated the positive effects of animal presence on the enhancement of memory and attention [37, 38] and improvements in children’s spatial abilities after dog and relaxation interventions have also been seen [39].

Companion animals may indirectly improve emotional regulation and cognition through improving executive function [34]. Maturational changes in executive function develop over the first two decades of life, meaning that infancy and childhood may be the optimal time for living with animals. Animals may also influence the development of self-regulation through stress regulation. As executive functions are negatively impacted by chronic stress but improved by social support [35], interaction with companion animals may also indirectly improve children’s ability to self-regulate and could contribute to the prevention of public health issues resulting from Early Adverse Childhood Experiences, however research has not supported this hypothesis so far [40].

Animal-owing infants and children may also benefit from a more linguistically stimulating environment, especially given that humans talk to pets in similar language used for small children (“motherese”) [41], potentially leading to enhanced or earlier development of speech. Interaction with animals during this developmental stage, and watching others interact with the animal, could also be hypothesized to enhance perspective-taking and prosocial behaviour [11]. Prosocial behaviour refers to actions intended to benefit others [42]. Attention to social cues like eye gaze direction has been shown to predict language outcome [43, 44]. During interaction with animals, where the reliance of verbal commands alone is rarely sufficient, social cues may be particularly important.

### Limited evidence for effects of pet ownership on childhood development

The findings of current research into the effects of companion animals are often difficult to interpret due to small samples, cross-sectional designs, lack of adjustment for known confounding factors such as socioeconomic variables, and often reducing complex and variable interactions with multiple species to simple animal presence/absence [9]. A previous systematic review [11] found that companion animal ownership and the significance of children’s bonds with them have been underexplored within the field of child development, particularly for the outcomes of cognitive and language development, and educational attainment. Further, there is a shortage of high quality and longitudinal studies that sufficiently controlled for confounding, which often attenuates positive effects of companion animals on child developmental outcomes [45, 46]. New well-designed research is needed using large, prospective datasets that examine the influence of different species which may be expected to be interacted with in different ways and intensities, whilst controlling for important confounding variables.

This study aims to address this research gap using a large dataset from the Avon Longitudinal Study of Parents and Children (ALSPAC) [47]. We investigated the associations between ‘pet ownership’ measured approximately every 18 months during childhood and adolescence, and a range of child developmental outcomes including anxiety, depression, self-esteem, behavioural difficulties, executive function, language development and educational attainment. Not only does this research make a novel contribution to the literature but the findings reported here can serve as a resource to parents as they make decisions about whether to introduce a new companion animal to the family.

Using examination of data from a longitudinal birth cohort, we hypothesised that companion animal ownership, in particular dog and cat ownership, would be:

- Positively associated with self-esteem, cognitive ability, language development and educational attainment.
- Negatively associated with anxiety, depression, and behavioural difficulties.

## Methods

### Participants

Pregnant women resident in Avon, UK with expected dates of delivery 1st April 1991 to 31st December 1992 were invited to take part in the study (Boyd et al., 2013; Fraser et al. 2013). The initial number of pregnancies enrolled was 14,541 (for these at least one questionnaire had been returned or a “Children in Focus” clinic had been attended by 19/07/99). Of these initial pregnancies, there was a total of 14,676 foetuses, resulting in 14,062 live births and 13,988 children who were alive at 1 year of age.

When the oldest children were approximately 7 years of age, an attempt was made to bolster the initial sample with eligible cases who had failed to join the study originally. As a result, when considering variables collected from the age of 7 onwards (and potentially abstracted from obstetric notes) there are data available for more than the 14,541 pregnancies mentioned above. The number of new pregnancies not in the initial sample (known as Phase I enrolment) that are additionally represented on the built files and reflecting enrolment status at the age of 24 is 913 (456, 262 and 195 recruited during Phases II, III and IV respectively). The phases of enrolment are described in more detail in the cohort profile paper (Boyd et al., 2013). The total sample size for analyses using any data collected after the age of seven is therefore 15,447 pregnancies, resulting in 15,589 foetuses. Of these 14,901 were alive at 1 year of age. 14,203 unique mothers were initially enrolled in the study. As a result of the additional phases of recruitment, a total of 14,833 unique women (G0 mothers) enrolled in ALSPAC as of September 2021. Further, 12,113 G0 partners have been in contact with the study by providing data and/or formally enrolling when this started in 2010. 3,807 G0 partners are currently enrolled.

A 10% sample of the ALSPAC cohort, known as the Children in Focus (CiF) group, attended clinics at the University of Bristol at various time intervals between 4 and 61 months of age in order to perform in-person measures for example practical assessments. The CiF group were chosen at random from the last 6 months of ALSPAC births (1432 families attended at least one clinic). Excluded were those mothers who had moved out of the area or were lost to follow-up, and those partaking in another study of infant development in Avon.

Compared to mothers in Avon and the rest of Great Britain, the ALSPAC cohort had a slight shortfall in the less affluent families (those living in rented accommodation, not having a car or being single or unmarried cohabiting), as well as a shortfall in ethnic minority mothers [48]. Children also had higher educational attainment at 16 years than the national sample and those lost to follow up had a lower educational attainment, plus children enrolled were more likely to be white and less likely to be eligible for free school meals than a national sample [47]. Those for whom ‘pet’ ownership was reported for the teenage years were more likely to be female with a higher maternal education level than those for whom ‘pet’ ownership was not reported [49].

### Measures

#### Pet measures

We will refer onwards to pet ownership rather than companion animals, as this is how the data were collected at the time. Pet data were collected approximately every 18 months. The pet ownership data from gestation up to age 18 years has been previously described in detail, please see [49, 50] for further information. In brief, pet ownership during gestation was available for 13,557 (96%) children as reported by caregivers, 7800 (97%) children at age 10 years, and retrospectively for 3098 (58%) adolescents at age 18 years, covering the 11–18 years period. Data included “do you have any pets?” and if so, how many cats, dogs, rabbits, rodents (mice, hamster, gerbil, etc.), birds (budgerigar, parrot, etc.), fish, tortoises/turtles, and after age 10, also horses. Parents or caregivers completed a postal questionnaire enquiring about the child’s activities when their child was typically (65%) aged 77 months (6 years) and reported if their child looked after a pet at home ‘Often’ (1342), ‘Occasionally’ (2946) or ‘Not at all’ (4045), which became our Pet Interaction (PI) variable.

To investigate the longitudinal impact of pet ownership duration, pet ownership history was constructed using two-step cluster analyses, an exploratory tool designed to reveal natural groupings (or clusters) within a dataset that would otherwise not be apparent. These typically encompassed ‘always, sometimes or never owned a pet’ up until that time point [49], which could then be used as an explanatory variable in its own right.

#### Outcome measures

The developmental outcome variables were derived from a variety of questionnaires completed by mothers/caregivers, or by children themselves, collected by post between age 2 and 15 years at specific time points, or from questionnaires or tasks conducted in Children in Focus (CiF) research clinics at specific ages (Table 1). They consisted of five broad areas of psychosocial development: (i) emotional health, (ii) behaviour, (iii) cognition, (iv) educational and (v) language development. Each area consisted of multiple endpoints (Table 1); more details about how specific measures were collected and analysed, including sample sizes for each outcome and corresponding pet ownership variable, are given in the Additional data file including Supplementary Figs. 1–11). Established cut-offs were used as described in the instrument validation where possible. Where no clinical cut-off score exists (such as for the RRS), cut-offs were calculated using standardized z scores, taking the highest (or lowest) tertile.

**Table 1:** ALSPAC outcomes and measures selected for the study (for how variables were analysed/coded see additional data file)

Outcome		Age (years)	Measure	Cross-sectional analyses (Pet ownership = Has any pet, has dog, has cat, has other pet)	Longitudinal analyses
Emotional Health	Self-esteem (scholastic competence)	8	Harter Self-Perception Profile (HSPP) (CiF clinic)	Pet ownership age 8	
	Self-esteem (global self worth)	8	Harter Self-Perception Profile (HSPP) (CiF clinic)	Pet ownership age 8	
	Separation anxiety	7, 10 and 13	Development And Wellbeing Assessment (DAWBA) (child-based questionnaire (parent reported))	Pet ownership age 7 Pet ownership age 10 Pet ownership age 13 Pet interaction age 6	Pet ownership repeated measures all time points. History of pet ownership by age 13
	Social anxiety	7, 10 and 13	Development And Wellbeing Assessment (DAWBA) (child-based questionnaire (parent reported))	Pet ownership age 7 Pet ownership age 10 Pet ownership age 13 Pet interaction age 6	Repeated measures all time points. History of pet ownership by age 13
	Generalised anxiety	7, 10 and 13	Development And Wellbeing Assessment (DAWBA) (child-based questionnaire (parent reported))	Pet ownership age 7 Pet ownership age 10 Pet ownership age 13 Pet interaction age 6	Pet ownership repeated measures all time points. History of pet ownership by age 13
	Depression	7	DAWBA (child-based questionnaire (parent reported))	Pet ownership age 7 Pet interaction age 6	
		10 and 13	Mood and Feelings Questionnaire (MFQ) (CiF clinic)	Pet ownership age 10 Pet ownership age13	History of pet ownership by age 13
Behaviour	Hyperactivity	3	Revised Rutter Scale (RRS) (parent reported)	Pet ownership age 3	
		11	Strengths and Difficulties Questionnaire (SDQ) (parent reported)	Pet ownership age 11	History of pet ownership by age 11
	Emotional symptoms	3	Revised Rutter Scale (RRS) (parent reported)	Pet ownership age 3	
		11	Strengths and Difficulties Questionnaire (SDQ) (parent reported)	Pet ownership age 11	History of pet ownership by age 11
	Conduct problems	3	Revised Rutter Scale (RRS)	Pet ownership age 3	
		11	Strengths and Difficulties Questionnaire (SDQ) (parent reported)	Pet ownership age 11	History of pet ownership by age 11
	Peer problems	11	Strengths and Difficulties Questionnaire (SDQ) (parent reported)	Pet ownership age 11	History of pet ownership by age 11
	Prosocial behaviour	3	Revised Rutter Scale (RRS) (parent reported)	Pet ownership age 3	
		11	Strengths and Difficulties Questionnaire (SDQ) (parent reported)	Pet ownership age 11	History of pet ownership by age 11
	Total behavioural difficulties	3	Revised Rutter Scale (RRS)	Pet ownership age 3	
		11	Strengths and Difficulties Questionnaire (SDQ) (parent reported)	Pet ownership age 11	History of pet ownership by age 11
Cognition/Executive function	Selective attention	8 and 11	Tests of Everyday Attention for Children (TEACh) (CiF clinic) - Sky Search	Pet ownership age 8, Pet ownership age 11	History of pet ownership by age 11
	Attentional Switching	8 and 11	Tests of Everyday Attention for Children (TEACh) (CiF clinic) - Dual Task	Pet ownership age 8 Pet ownership age 11	Pet ownership repeated measures all time points. History of pet ownership by age 11.
	Attentional control	8 and 11	Tests of Everyday Attention for Children (TEACh) (CiF clinic) - Same worlds	Pet ownership age 8 Pet ownership age 11	History of pet ownership by age 11.
		8 and 11	Tests of Everyday Attention for Children (TEACh) (CiF clinic) - Opposite worlds	Pet ownership age 8 Pet ownership age 11	History of pet ownership by age 11
	Inhibition/impulsivity	10	Stop-signal task (CiF clinic) (150ms delay)	Pet ownership age 10	
		10	Stop-signal task (CiF clinic) (250ms delay)	Pet ownership age 10	
	Working memory	8	Digit recall (CiF clinic)	Pet ownership age 8	
		10	Counting span task (CiF clinic)	Pet ownership age 10	
Education	Reading	7	Standard Assessment Test (SATs) Key Stage 1 (KS1)	Pet ownership age 7	
	Writing	7	Standard Assessment Test (SATs) Key Stage 1 (KS1)	Pet ownership age 7	
	Maths	7	Standard Assessment Test (SATs) Key Stage 1 (KS1)	Pet ownership age 7	
	Summary score	7	Standard Assessment Test (SATs) Key Stage 1 (KS1)	Pet ownership age 7	
	English	11	Standard Assessment Test (SATs) Key Stage 2 (KS)	Pet ownership age 11	
	Maths		Standard Assessment Test (SATs) Key Stage 2 (KS)	Pet ownership age 11	
	Science		Standard Assessment Test (SATs) Key Stage 2 (KS)	Pet ownership age 11	
	Summary score		Standard Assessment Test (SATs) Key Stage 2 (KS)	Pet ownership age 11	
	English	15	General Certificate of Secondary Education (GCSE) attainment grades	Pet ownership age 15	History of pet ownership by age 15
	Maths	15	General Certificate of Secondary Education (GCSE) attainment grades	Pet ownership age 15	History of pet ownership by age 15
	Biological Sciences	15	General Certificate of Secondary Education (GCSE) attainment grades	Pet ownership age 15	History of pet ownership by age 15
	Chemistry	15	General Certificate of Secondary Education (GCSE) attainment grades	Pet ownership age 15	History of pet ownership by age 15
	Physics	15	General Certificate of Secondary Education (GCSE) attainment grades	Pet ownership age 15	History of pet ownership by age 15
	Achieved 5 A*-C	15	General Certificate of Secondary Education (GCSE) attainment grades	Pet ownership age 15	History of pet ownership by age 15
Language	Language development score	2 and 5	Reynell Development Language Scale (RDLS) (CiF clinic)	Pet ownership age 2 Pet ownership age 5	
	Total Communication Score	2	MacArthur Communicative Development Inventories (MCDI) (parent reported)	Pet ownership age 2	
	Vocabulary score	2	MacArthur Communicative Development Inventories (MCDI) (parent reported)	Pet ownership age 2	
	Non-verbal communication score	2	MacArthur Communicative Development Inventories (MCDI) (parent reported)	Pet ownership age 2	
	Social development score	2	MacArthur Communicative Development Inventories (MCDI) (parent reported)	Pet ownership age 2	

Please note that the study website contains details of all the data that is available through a fully searchable data dictionary and variable search tool at http://www.bristol.ac.uk/alspac/researchers/our-data/.

#### Confounding variables

Considering that the apparent associations between key variables of interest and the effects of owning pets may be influenced by numerous confounding factors, a large range of variables were included within the project dataset. Final confounders in each model were chosen on the basis of previous analyses that have found factors associated with PO in this cohort [49, 50], theoretical plausibility and based on what we know are associated with the predictor and outcome variables from prior research. Potential confounding measures adjusted for (see Additional File) included sex, ethnicity, maternal mental health, socioeconomic status, family factors (older children living with the child, whether the child has a twin, child attended day care, and parental marital status), and child factors (developmental delay (Denver development scale [51]), child temperament (Toddler Temperament Scale [52]), and stressful life events (maternal questionnaire [53]). DAGitty software was used to build causal models for each developmental outcome based on the concept of directed acyclic graphs (see Supplementary Figs. 12–14) and by selecting confounders based on empirical evidence from previous studies. The primary theoretical considerations when considering confounder selection are described below.

Detecting gender differences in pet ownership is difficult to do using only family pet ownership data, as many families have both male and female children [54]. Nonetheless, sex of the study child was controlled for within all analyses as there are likely to be differences according to developmental outcome. Girls have indicated higher rates of ownership of pets in datasets including ALSPAC [50, 55].

As ALSPAC is not very ethnically diverse, ethnicity is not usually used as a confounder. It was however, controlled for in the language analyses. Previous research has found that compared with non-owners, dog and/or cat owners were more likely to be of Caucasian ethnicity and have better English language skills [46].

Maternal mental health is frequently used as a confounding factor in many ASLPAC developmental studies [56, 57]. It can be hypothesised that experiencing poor mental health may impact decisions to own pets, especially in childhood as the parent is the one making the pet-owning decisions. All models were adjusted for maternal depression (Edinburgh Postnatal Depression Scale EPDS [58] dichotomized at a cut-off of 13), and maternal anxiety (Crown-Crisp Experiential Index CCI [59] dichotomized at a cut-off of 9).

Again, it is to be expected that family demographic factors such as socio-economic status exert some influence on the likelihood of acquisition of pets, as they affect the parent who makes the pet-owning decisions. Socioeconomic status is an important consideration when looking at the pet effect on child development. Previous research has found that compared with non-owners, dog and/or cat owners were significantly less likely to have a child who received free or reduced lunch at school, have higher monthly housing costs, worked more hours per week, and more likely to live in a house [46]. Other research has found that dog ownership in the general population decreases as years of education or family affluence level increases [60–62]. Supporting this, another study found dog ownership in children was associated with higher levels of deprivation [63]. In ALSPAC, throughout childhood and adolescence, professional occupations were least likely to own pets [49, 50].

Confounding measures of socioeconomic status included highest parental social class (occupational social class 1991 British Office of Population and Census Statistics [64] classification), maternal education (coded on a five-level scale, with the lowest score indicating lowest educational attainment Certificate of Secondary Education (CSE) or vocational qualification and the highest level indicating university degree), grouped maternal age at delivery (< 21, 21–30, > 30), overcrowding (> 5 people), house type (detached, semi-detached, end-terrace, terraced, flat/room in someone else’s house/other), financial difficulties (occurrence of major financial problems since pregnancy versus none), ownership of home (owned accommodation; privately rented; subsidized housing), and housing defects, family income and car access (these variables were mainly derived from questionnaires administered during the antenatal period).

Families who choose to own companion animals may be different to families without companion animals in terms of the ‘social climate’. Parenting practices and parental bonding effects on the development of children are difficult to detangle from the contribution made by companion animals [65]. Research has found that not only are families with children more likely to have pets [66], but that pet ownership reaches a peak in families with children at middle childhood, between 8 and 12 years [49, 54]. The absence of any or younger siblings can also influence attachment to the pet [55]. Where appropriate, models were adjusted for older children living with the child, whether the child has a twin, if the child attends day care, and parental marital status (ALSPAC maternally reported questionnaires [53]).

Lastly, there are child factors that are important to adjust for when exploring associations between PO and developmental outcomes. These factors are rarely accounted for in previous studies looking at the impact of pets on human development. It is plausible to suggest that children with developmental or temperamental difficulties, and those who have been through stressful live events, may be more likely to own pets, as parents often obtain pets in attempt to relieve their child’s difficulties or to hone their social and emotional development [67]. Where appropriate, models have been adjusted for developmental delay (Denver development scale [51]), child temperament (Toddler Temperament Scale [52]), and stressful life events (ALSPAC maternally reported questionnaires [53]).

### Analysis

Univariable and multivariable linear or logistic regression analyses examined associations between ownership of any pets, dog, cat and other/miscellaneous pets, (or history of pet/dog ownership), and developmental outcomes for each age at which the outcome was measured (See Table 1).

Cross-sectional and longitudinal analyses were conducted, depending on the availability of longitudinal measures. As outlined in Table 1, due to cohort design and assessment schedules in ALSPAC, longitudinal data is not available for all measures, while testing intervals for exposure and outcome were not always identical. Where repeated measures were available, associations were assessed using random effect hierarchical regression models in MLwiN version 3.02, to account for clustering of data within individuals across all time points available. The majority of analyses were based on contemporaneous associations of variables, as the data did not allow for lag analyses due to different outcome measures being used at different time points. However, the longer-term longitudinal impact of pet ownership duration on development was also examined using the pet ownership history variables (excluding language development at young ages).

Final covariate selection for the adjustment in multivariable modelling was also based on statistical considerations as well as the causal models previously described. Variables remained in the model if there was considerable wider evidence to support their inclusion for adjustment, e.g., factors associated with pet ownership [49, 50], good evidence for an association (*P* < .05) in this model, or if their removal (by backwards stepwise) resulted in substantial change to the effect of other variables (10% or greater). This method resulted in most confounding variables being included in each model; variables which were and were not included are described in the legend to each table.

To address biases from attrition, missing data for all confounders in the models were imputed using multiple imputation by chained equations (MICE) [68] in SPSS Version 24 using an MCMC (Markov chain Monte Carlo) algorithm known as fully conditional specification or chained equations imputation (see Additional File for more information). Exposure and outcome variables were not imputed. A more detailed description of missing data for each confounder at each time point can be found in Additional File Supplementary Table 1.

Considering the number of multiple comparisons made within the study, reducing the *P* value criterion (Bonferroni correction) was considered in order to reduce the chance of Type 1 error. However, the Bonferroni correction addresses the Type 1 error problem at the expense of Type 2 error, thus severely reducing power to detect an important effect [69]. To remain cautious, we present patterns of results, and ‘weak’ or ‘strong’ evidence of an association instead of stating statistical significance or relying on the magnitude of *P* values [70], in line with ALSPAC Committee recommendations. In addition, as advised by other scholars [71] *P* values are presented but not in isolation and effect estimates are also provided. As the pet interaction variable was collected only once (age 6 years), the developmental outcomes that could be explored for association were: anxiety and depression at age 7 years.

## Results

Pet ownership increased from 58% during gestation to 74% at 10 years and 72% by age 18 [49]. At age 10 years, cat ownership was 31% and dog ownership was 26%. Similarly, the frequency of owning fish, rodents and rabbits increased until age 11 years, but then declined. Cats were the most commonly reported pet up to age 15 years; dogs were the most common pet type among older adolescents, rising to 37% by 18 years. Findings from complete case analyses were very similar to the imputed missing data analyses thus only the latter are presented. Analyses model numbers using multiple imputation varied from *n* = 393–8963 (see Additional File Supplementary Figs. 1–11).

### Emotional health

Prevalence of child emotional health outcomes ranged from 4% (separation anxiety aged 13) to 23.9% (low scholastic competence aged 8) (for more details see Additional File Supplementary Tables 2–3). At age 8, children who owned any pets (OR = 0.85, 95%ci-0.73–0.98, p= ·026) or cats (OR = 0.83, 95%CI = 0.73–0.95, p= ·006) had lower odds of concurrent high self-esteem (scholastic competence). In contrast, there was no evidence of an association between pet ownership and self-esteem (global self-worth) at age 8 (Table 2). At age 7, there was weak evidence of an association between owning any pets (OR = 1.31, 95%CI = 1.03–1.67, p= ·027) or owning ‘other/miscellaneous’ pets (OR = 1.28, 95%CI = 1.04–1.57, p= ·021) and higher odds of social anxiety measured concurrently (Table 2). However, the association with owning any pets did not remain during longitudinal analyses after accounting for scores at all three time points (7, 10, 13) within a repeated measures model (b = 0.09, 95%CI=-0.29-0.49, *p* = .621) (Additional file Supplementary Table 4). There was no evidence of an association between pet ownership and separation anxiety, depression, anxious and depressive symptoms, or emotional health difficulties at either age 7, 10 or 13 (Table 2; Additional file Supplementary Table 5) nor if considering longitudinal analysis using pet ownership history at 13 years (Additional file Supplementary Table 6) or pet interaction at age 6 (Additional file Supplementary Table 7).

**Table 2:** Emotional health results showing: Univariable and multivariable associations between pet ownership at age 8 and the likelihood of low self-esteem, as measured with the Harter self-perception profile subscales (scholastic competence and global self-worth) at age 8; Univariable and multivariable associations between pet ownership at ages 7, 10 and 13 and DAWBA (Development and Wellbeing Assessment) separation anxiety, social anxiety, and generalized anxiety disorder symptoms at ages 7, 10 and 13 years. For depression findings please see additional file supplementary table 5

	Emotional health outcome		Univariable		Multivariable (adjusted)	
Age		*N*	OR (95% *CI*)	*p*	OR (95% *CI*)	*p*
8	**Self esteem - Scholastic Competence (low score** ≤ 14)					
	Has any Pet	3951	0.81 (0.71, 0.92)	0.006*	0.85 (0.73, 0.98)	0.026*
	Has Dog	3952	0.79 (0.68, 0.92)	0.002*	0.86 (0.74, 1.00)	0.054
	Has Cat	3952	0.80 (0.71, 0.92)	0.001*	0.83 (0.73, 0.95)	0.006*
	Has other/miscellaneous pets	3952	0.92 (0.81, 1.04)	0.197	0.94 (0.83, 1.07)	0.323
8	**Self esteem - Global self-worth (low score** ≤ 16)					
	Has any Pet	3938	0.91 (0.78, 1.05)	0.199	0.93 (0.79, 1.09)	0.368
	Has Dog	3939	0.97 (0.83, 1.14)	0.747	1.05 (0.89, 1.24)	0.544
	Has Cat	3939	0.92 (0.80, 1.06)	0.244	0.94 (0.81, 1.08)	0.374
	Has other /miscellaneous pets	3939	0.94 (0.82, 1.07)	0.338	0.95 (0.83, 1.09)	0.451
	**Separation Anxiety (**any separation anxiety symptom(s) “a lot more than others”)					
	Has any Pet					
7		6638	1.01 (0.82, 1.24)	0.945	0.99 (0.79, 1.22)	0.895
10		6375	1.15 (0.91, 1.46)	0.230	1.16 (0.91, 1.48)	0.220
13		2387	1.24 (0.69, 2.20)	0.465	1.15 (0.64, 2.07)	0.639
	Has Dog					
7		6635	0.96 (0.76, 1.22)	0.778	0.95 (0.75, 1.21)	0.692
10		6374	1.11 (0.88, 1.39)	0.376	1.12 (0.88, 1.41)	0.365
13		2390	1.30 (0.80, 2.11)	0.289	1.18 (0.71, 1.94)	0.525
	Has Cat					
7		6634	1.27 (0.95, 1.43)	0.132	1.11 (0.91, 1.36)	0.312
10		6374	1.22 (0.98, 1.50)	0.069	1.18 (0.95, 1.46)	0.138
13		2391	1.57 (0.98, 2.50)	0.059	1.49 (0.93, 2.40)	0.101
	Has other/miscellaneous pets					
7		6635	1.04 (0.86, 1.26)	0.688	1.04 (0.85, 1.26)	0.714
10		6374	1.02 (0.83, 1.24)	0.886	1.03 (0.84, 1.27)	0.776
13		2389	0.86 (0.54, 1.38)	0.545	0.83 (0.52, 1.34)	0.450
	**Social Anxiety (any social fears “a lot”)**					
	Has any Pet					
7		7208	1.28 (1.01, 1.62)	0.040*	1.31 (1.03, 1.67)	0.027*
10		6714	1.23 (0.97, 1.55)	0.092	1.25 (0.98, 1.59)	0.068
13		2604	1.44 (0.99, 2.09)	0.056	1.30 (0.89, 1.90)	0.178
	Has Dog					
7		7204	0.91 (0.70, 1.17)	0.467	0.92 (0.71, 1.19)	0.529
10		6713	1.04 (0.83, 1.30)	0.724	1.05 (0.83, 1.33)	0.679
13		2608	1.10 (0.80, 1.51)	0.533	0.97 (0.70, 1.34)	0.847
	Has Cat					
7		7203	1.18 (0.95, 1.46)	0.131	1.15 (0.92, 1.42)	0.221
10		6713	1.13 (0.91, 1.39)	0.257	1.10 (0.89, 1.36)	0.382
13		2609	1.08 (0.79, 1.47)	0.627	1.05 (0.77, 1.44)	0.771
	Has other/miscellaneous pets					
7		7204	1.24 (1.01, 1.52)	0.035*	1.28 (1.04, 1.57)	0.021*
10		6713	1.10 (0.90, 1.35)	0.326	1.14 (0.93, 1.39)	0.197
13		2607	1.06 (0.79, 1.41)	0.713	0.99 (0.73, 1.33)	0.920
	**Generalized Anxiety (any of the worries “often”)**					
	Has any Pet					
7		7244	1.05 (0.87, 1.27)	0.568	1.06 (0.88, 1.28)	0.544
10		2890	1.03 (0.83, 1.27)	0.820	1.01 (0.81, 1.26)	0.933
13		1126	1.22 (0.84, 1.76)	0.294	1.13 (0.77, 1.66)	0.524
	Has Dog					
7		7240	0.98 (0.80, 1.20)	0.872	0.98 (0.79, 1.20)	0.828
10		2889	1.19 (0.97, 1.47)	0.089	1.17 (0.95, 1.46)	0.145
13		1129	1.10 (0.79, 1.53)	0.547	0.98 (0.69, 1.38)	0.891
	Has Cat					
7		7239	1.19 ( 0.99, 1.41)	0.055	1.12 (0.94, 1.34)	0.205
10		2889	1.25 (1.03, 1.51)	0.022*	1.19 (0.99, 1.46)	0.068
13		1130	1.14 (0.83, 1.55)	0.419	1.08 (0.78, 1.50)	0.647
	Has other/miscellaneous pets					
7		7240	1.07 (0.91, 1.27)	0.379	1.11 (0.94, 1.32)	0.215
10		2889	1.05 (0.87, 1.26)	0.632	1.02 (0.85, 1.24)	0.803
13		1128	1.05 (0.78, 1.42)	0.735	1.02 (0.75, 1.40)	0.890

*P < .05

Analyses of self-esteem measures adjusted for: sex, maternal depression measured at child age 8, maternal anxiety measured at child age 6, overcrowding (child age 8), house type (child age 7), highest parental social class (antenatal period), maternal education (antenatal period), maternal age at delivery (antenatal period), financial difficulties (antenatal period), home ownership status (antenatal period), car ownership (antenatal period), older children living with child (antenatal period), developmental delay measured at child age 30 months old, IQ measured at child age 8 years (accounted for in scholastic competence only), stressful life events measured at child age 4 years and maternal bonding measured at child age 3

Analyses of anxiety measures adjusted for: sex, maternal depression measured at child age 8 and 11 years, maternal anxiety measured at child age 6 and 11 years, overcrowding (child age 7, 8 and 10 years), house type (child age 7 and 10 years), highest parental social class (antenatal period), maternal education (antenatal period), maternal age at delivery (antenatal period), financial difficulties (antenatal period), home ownership status (antenatal period), and car ownership (antenatal period), developmental delay measured at child age 30 months, older children living with child, stressful life events at child age 3, 9 and 11 years and maternal bonding measured at child age 3 years

Results did not differ when accounting for parental marital status, child temperament and dog walking (Number of times in typical week respondent walked or jogged with household dog(s)), therefore these variables were discarded from the final models

### Behavioural development

Prevalence of child behavioural difficulties by our cut off method (note this is not a clinical diagnosis) ranged from 7% (prosocial difficulties aged 11) to 57% (conduct difficulties aged 3) (Additional file Supplementary Table 8). There was no evidence of an association between pet ownership at ages 3 or 11 and concurrently measured emotional health difficulties (Table 3). At age 3, there was weak evidence of an association between owning cats and higher odds of hyperactivity (OR = 1.12, 95%CI = 1.01–1.24, *p* = .037). Owning pets (OR = 1.14, 95%CI = 1.05–1.25; *p* = .003) and cats (OR = 1.17, 95%CI = 1.06–1.29, *p* = .001) at age 3, and dogs at ages 3 (OR = 1.19, 95%CI = 1.07–1.33, *p* = .002) and 11 (OR = 1.44, 95%CI = 1.11–1.86, *p* = .006) were more strongly associated with a higher likelihood of conduct problems at these ages. Owning ‘other/miscellaneous’ pets at age 3 was associated with lower likelihood of experiencing prosocial difficulties at that age (OR = 0.88, 95%CI = 0.79–0.97, *p* = .012). When prosocial behaviour analyses were repeated using linear regression, we found that owning a dog at age 3 was associated with a higher concurrent prosocial behaviour score (b = 0.24, 95%=0.06–0.41, *p* = .004), as was owning other/miscellaneous pets (b = 0.18, 95%CI = 0.03–0.33, *p* = .021). At age 11, owning ‘other/miscellaneous’ pets wesre associated with a lower likelihood of peer problems (b = 0.72, 95%CI-0.57–0.89, *p* = .004) and weakly a lower likelihood of total behavioural difficulties (b = 0.73, 95%CI-0.53–0.99, *p* = .044) at that age. There was no evidence of an association during longitudinal analyses of pet ownership history (sometimes or always owned pets up to 11 years) and behavioural difficulties symptoms at 11 years (Additional file Supplementary data Table 9).

**Table 3:** Univariable and multivariable associations between pet ownership at ages 3 and 11 years and behavioural difficulties and ages 3 and 11 years. Two different measures, using the same subscales, but different scoring methods, were used at age 3 (revised Rutter score) and at age 11 (strengths and difficulties Questionnaire)

			Univariable		Multivariable (adjusted)	
Age		*N*	OR (95% *CI*)	*p*	OR (95% *CI*)	*p*
	**Emotional difficulties**					
	Has any Pet					
3		8980	0.94 (0.86, 1.02)	0.127	0.95 (0.87, 1.04)	0.265
11		2642	1.17 (0.86, 1.58)	0.307	0.98 (0.71, 1.34)	0.886
	Has Dog					
3		8973	0.97 (0.88, 1.08)	0.627	0.97 (0.87, 1.08)	0.545
11		2645	1.16 (0.89, 1.51)	0.258	1.08 (0.82, 1.42)	0.586
	Has Cat					
3		8973	0.95 (0.87, 1.05)	0.326	0.96 (0.87, 1.05)	0.359
11		2646	1.09 (0.86, 1.41)	0.459	0.99 (0.76, 1.28)	0.925
	Has other/miscellaneous pets					
3		8973	0.89 (0.82, 0.98)	0.017*	0.92 (0.84, 1.02)	0.097
11		2645	1.03 (0.81, 1.31)	0.799	0.91 (0.71, 1.17)	0.479
	**Hyperactivity**					
	Has any Pet					
3		7789	1.05 (0.96, 1.15)	0.278	1.09 (0.99, 1.21)	0.057
11		2640	1.12 (0.78, 1.60)	0.535	1.28 (0.87, 1.86)	0.207
	Has Dog					
3		7783	1.03 (0.92, 1.15)	0.670	1.05 (0.92, 1.17)	0.464
11		2643	1.18 (0.86, 1.60)	0.309	1.22 (0.88, 1.69)	0.231
	Has Cat					
3		7783	1.09 (0.98, 1.20)	0.095	1.12 (1.01, 1.24)	0.037*
11		2644	1.19 (0.88, 1.60)	0.243	1.21 (0.89, 1.65)	0.221
	Has other/miscellaneous pets					
3		7783	0.96 (0.87, 1.06)	0.396	1.00 (0.91, 1.11)	0.954
11		2643	0.73 (0.55, 0.97)	0.029*	0.78 (0.58, 1.05)	0.103
	**Conduct Difficulties**					
	Has any Pet					
3		8980	1.14 (1.05, 1.24)	0.002*	1.14 (1.05, 1.25)	0.003*
11		2643	1.38 (1.02, 1.86)	0.040*	1.29 (0.94, 1.78)	0.111
	Has Dog					
3		8973	1.18 (1.06, 1.31)	0.002*	1.19 (1.07, 1.33)	0.002*
11		2646	1.47 (1.15, 1.88)	0.002*	1.44 (1.11, 1.86)	0.006*
	Has Cat					
3		8973	1.16 (1.06, 1.27)	0.002*	1.17 (1.06, 1.29)	0.001*
11		2647	1.17 (0.93, 1.49)	0.188	1.09 (0.85, 1.39)	0.509
	Has other/miscellaneous pets					
3		8973	1.04 (0.95, 1.14)	0.365	1.01 (0.92, 1.11)	0.851
11		2646	0.90 (0.72, 1.14)	0.381	0.85 (0.66, 1.08)	0.173
	**Peer Problems**					
11	Has any Pet	2644	0.93 (0.72, 1.20)	0.574	0.93 (0.71, 1.22)	0.593
	Has Dog	2647	1.15 (0.91, 1.45)	0.233	1.16 (0.91, 1.49)	0.234
	Has Cat	2648	1.19 (0.96, 1.49)	0.113	1.17 (0.93, 1.47)	0.192
	Has other/miscellaneous pets	2647	0.73 (0.59, 0.91)	0.004*	0.72 (0.57, 0.89)	0.004*
	**Prosocial**					
	Has any Pet					
3		7944	0.93 (0.85, 1.01)	0.098	0.95 (0.86, 1.04)	0.261
11		2645	0.84 (0.57, 1.23)	0.371	0.91 (0.61, 1.37)	0.656
	Has Dog					
3		7937	0.89 (0.79, 0.99)	0.033*	0.89 (0.79, 1.01)	0.068
11		2648	1.20 (0.84, 1.72)	0.309	1.37 (0.94, 1.99)	0.103
	Has Cat					
3		7937	1.01 (0.91, 1.11)	0.885	1.02 (0.92, 1.13)	0.737
11		2649	0.96 (0.67, 1.36)	0.803	0.95 (0.66, 1.37)	0.795
	Has other/miscellaneous pets					
3		7937	0.87 (0.79, 0.96)	0.004*	0.88 (0.79, 0.97)	0.012*
11		2648	0.76 (0.55, 1.06)	0.109	0.79 (0.57, 1.13)	0.202
	**Total Behavioural Difficulties**					
	Has any Pet					
3		8963	1.04 (0.95, 1.13)	0.435	1.07 (0.97, 1.17)	0.167
11		2643	1.23 (0.84, 1.79)	0.282	1.15 (0.77, 1.72)	0.506
	Has Dog					
3		8956	1.04 (0.94, 1.16)	0.465	1.05 (0.93, 1.17)	0.450
11		2646	1.33 (0.97, 1.82)	0.079	1.29 (0.92, 1.80)	0.141
	Has Cat					
3		8956	1.06 (0.96, 1.16)	0.251	1.07 (0.97, 1.19)	0.181
11		2647	1.29 (0.96, 1.75)	0.090	1.21 (0.88, 1.66)	0.242
	Has other/miscellaneous pets					
3		8956	0.95 (0.87, 1.04)	0.294	0.98 (0.88, 1.08)	0.615
11		2646	0.79 (0.59, 1.06)	0.116	0.73 (0.53, 0.99)	0.044*

*P < .05

Analyses adjusted for: sex, birthweight, maternal depression measured at child age 2 and 11 years, maternal anxiety measured at child age 2 and 11 years, overcrowding (child age 2 and 10 years), highest parental social class (antenatal period), maternal education (antenatal period), maternal age at delivery (antenatal period), family income (antenatal period), housing defects (antenatal period), financial difficulties (antenatal period), home ownership status (antenatal period), car ownership (antenatal period), developmental delay measured at child age 30 months, child temperament at 2 years, older children living with child (antenatal period), stressful life events at child age 2 and 11 years and maternal bonding measured at child age 3 years

Results did not differ when accounting for house type or dog walking (Number of times in typical week respondent walked or jogged with household dog(s)), therefore these variables were discarded from the final model

### Cognitive development

At ages 8 and 11 there was no strong cross-sectional evidence of an association between pet ownership and measures of selective attention, attentional control (Table 4), impulsivity or working memory (Additional file Supplementary Table 10). However, owning a dog was associated with scoring 0.42 points (95%CI = 0.12–0.17, *p* = .005) higher (indicating poorer ability) in attentional switching only at age 11. This association remained during longitudinal analyses accounting for scores at both time points within a repeated measures model (b = 0.67, 95%CI = 0.25–1.08, *p* = .001). There was no evidence of a longitudinal association between pet ownership history and cognitive performance at 11 years (Additional file Supplementary Table 11).

**Table 4:** Univariable and multivariable associations between pet ownership at ages 8, 10 and 11, and attention. For impulsivity and working memory please see additional file supplementary table 10

			Univariable		Multivariable (adjusted)	
Age		*N*	b (95% *CI*)	*p*	b (95% *CI*)	*p*
	**Selective Attention**					
	**Sky Search Task (score)**					
	Has any Pet					
8		5720	-0.02 (-0.13, 0.09)	0.693	0.03 (-0.08, 0.14)	0.577
11		2522	-0.02 (-0.13, 0.08)	0.682	0.09 (-0.02, 0.19)	0.100
	Has Dog					
8		5721	0.05 (-0.06, 0.168)	0.352	0.05 (-0.06, 0.16)	0.388
11		2524	0.02 (-0.08, 0.12)	0.687	0.06 (-0.04, 0.16)	0.209
	Has Cat					
8		5721	-0.03 (-0.13, 0.08)	0.606	-0.01 (-0.10, 0.09)	0.983
11		2525	-0.08 (-0.16, 0.01)	0.094	-0.04 (-0.13, 0.05)	0.399
	Has other/miscellaneous pets					
8		5721	-0.07 (-0.17, 0.02)	0.132	-0.00 (-0.09, 0.08)	0.947
11		2524	-0.09 (-0.17, 0.00)	0.051	-0.03 (-0.13, 0.06)	0.490
	**Attentional Switching (Dual Task) (score)**					
	Has any Pet					
8		5228	0.32 (-0.72, 1.35)	0.548	0.71 (-0.32, 1.75)	0.178
11		2357	0.15 (-0.15, 0.46)	0.326	0.27 (-0.05, 0.59)	0.098
	Has Dog					
8		5228	0.46 (-0.64, 1.55)	0.414	0.46 (-0.65, 1.56)	0.417
11		2359	0.39 (0.10, 0.67)	0.007*	0.42 (0.12, 0.71)	0.005*
	Has Cat					
8		5228	0.49 (-0.48, 1.47)	0.325	0.65 (-0.33, 1.62)	0.192
11		2360	0.01 (-0.26, 0.27)	0.950	0.05 (-0.22, 0.32)	0.715
	Has other/miscellaneous pets					
8		5228	-0.35 (-1.25, 0.56)	0.459	-0.06 (-0.97, 0.85)	0.894
11		2359	-0.08 (-0.33, 0.17)	0.559	-0.01 (-0.26, 0.25)	0.991
	**Attentional Control**					
	**Same worlds task (time seconds)**					
	Has any Pet					
8		5745	-0.06 (-0.25, 0.12)	0.477	-0.03 (-0.21, 0.16)	0.773
11		2448	-0.03 (-0.20, 0.14)	0.715	0.03 (-0.14, 0.20)	0.723
	Has Dog					
8		5746	0.18 (-0.01, 0.38)	0.055	0.16 (-0.04, 0.35)	0.111
11		2450	0.09 (-0.06, 0.25)	0.236	0.07 (-0.09, 0.23)	0.376
	Has Cat					
8		5746	-0.02 (-0.19, 0.16)	0.867	-0.01 (-0.17, 0.17)	0.966
11		2451	0.01 (-0.14, 0.15)	0.910	0.02 (-0.12, 0.17)	0.778
	Has other/miscellaneous pets					
8		5746	-0.13 (-0.28, 0.04)	0.126	-0.09 (-0.25, 0.07)	0.268
11		2450	-0.13 (-0.26, 0.01)	0.071	-0.08 (-0.22, 0.06)	0.250
	**Opposite worlds task (time seconds)**					
	Has any Pet					
8		5739	-0.33 (-0.67, 0.02)	0.068	-0.27 (-0.63, 0.08)	0.126
11		2446	0.01 (-0.22, 0.25)	0.911	0.14 (-0.09, 0.38)	0.232
	Has Dog					
8		5740	0.23 (-0.14, 0.59)	0.231	0.16 (-0.21, 0.53)	0.400
11		2448	0.11 (-0.10, 0.32)	0.303	0.09 (-0.12, 0.31)	0.389
	Has Cat					
8		5740	-0.07 (-0.40, 0.26)	0.686	-0.05 (-0.38, 0.28)	0.764
11		2449	0.06 (-0.14, 0.25)	0.580	0.11 (-0.09, 0.30)	0.282
	Has other/miscellaneous pets					
8		5740	-0.31 (-0.62, -0.01)	0.047*	-0.26 (-0.56, 0.05)	0.105
11		2448	-0.19 (-0.37, 0.00)	0.050*	-0.11 (-0.29, 0.08)	0.268

*P < .05

Analyses were adjusted for: sex, maternal depression at ages 8 and 11 years, maternal anxiety at ages 6 and 11 years, overcrowding at 8 and 10 years, house type at ages 7 and 10 years, highest parental social class (antenatal period), maternal education (antenatal period), maternal age at delivery (antenatal period), home ownership status (antenatal period), family income (antenatal period) and car ownership (antenatal period), birthweight, developmental delay measured at child age 30 months, child temperament at 2 years, older children living in the house (antenatal period), stressful life events at almost 4 years, 9 and 11 years old, and mother-child bonding at child age 3 years

Results did not differ when accounting for parental marital status and financial difficulties therefore these variables were discarded from the final models

### Educational development

Key Stage 1 (KS1) and Key Stage 2 (KS2) level attainment within ALSPAC were equivalent to national averages, however, children in ALSPAC had a higher GCSE attainment at the age of 16 years in comparison to the National Pupil Database (NPD) ‘Key Stage 4’ (KS4) national sample dataset records (Boyd et al., 2013).

Children who owned pets, dogs and other pets at age 6–7 years (KS1) scored 0.09, (95% CI-0.17- -0.02), *p* = .012), 0.17, (95% CI=-0.25- -0.08), *p* < .001) and 0.12, (95% CI=-0.l9- -0.06, *p* < .001) points respectively lower in reading scores at this stage (Table 5). Similarly, they scored lower in writing (pets b=-0.13, 95% CI= -0.19- -0.06, *p* = .001; dogs b=-0.15, 95% CI=-0.22- -0.08, *p* < .001; other pets b=-0.12, 95% CI=-0.17- -0.06, *p* < .001), maths (pets b= -0.07, 95% CI=-0.14- -0.00, *p* = .041; dogs b= -0.09, 95% CI=-0.17- -0.02, *p* = .015; other pets b=-0.08, 95% CI=-0.14- -0.02, *p* = .013) and total Standard Assessment Tests (SAT) summary scores (pets b=-0.29, 95% CI=-0.47- -0.11, *p* = .002; dogs b=-0.41, 95% CI=-0.61- -0.21, *p* < .001; other pets b=-0.32, 95% CI=-0.48- -0.15, *p* < .001) (Table 5). Although the evidence for these associations appears strong (*P* < .001) it is worth noting, however, that effect sizes were very small (range. 0.07–0.41 point decrease in score for owning the type of pet) and may not be clinically significant. There was consistently no evidence that ownership of a cat at this stage was associated with KS1 SAT attainment (Table 5).

**Table 5:** Univariable and multivariable associations between pet ownership at ages 7, 11 and 15, and Key Stage 1 (KS1), Key Stage 2 (KS2) and General Certificate of Secondary Education (GCSE) attainment

		Univariable		Multivariable (adjusted)	
KS1					
Subject	*N*	B (95% *CI*)	*p*	B (95% *CI*)	*p*
**Reading (score)**					
Has any Pet	5760	-0.20 (-0.28, -0.11)	< 0.001*	-0.09 (-0.17, -0.02)	0.012*
Has Dog	5755	-0.36 (-0.46, -0.27)	< 0.001*	-0.17 (-0.25, -0.08)	< 0.001*
Has Cat	5755	-0.05 (-0.14, 0.03)	0.228	-0.01 (-0.09, 0.06)	0.726
Has other/miscellaneous pets	5755	-0.19 (-0.27, -0.12)	< 0.001*	-0.12 (-0.19, -0.06)	< 0.001*
**Writing (score)**					
Has any Pet	5762	-0.21 (-0.29, -0.14)	< 0.001*	-0.13 (-0.19, -0.06)	< 0.001*
Has Dog	5757	-0.31 (-0.39, -0.23)	< 0.001*	-0.15 (-0.22, -0.08)	< 0.001*
Has Cat	5757	-0.08 (-0.15, -0.01)	0.035*	-0.04 (-0.10, 0.02)	0.170
Has other/miscellaneous pets	5757	-0.16 (-0.23, -0.09)	< 0.001*	-0.12 (-0.17, -0.06)	< 0.001*
**Maths (score)**					
Has any Pet	5754	-0.16 (-0.24, -0.08)	< 0.001*	-0.07 (-0.14, -0.00)	0.041*
Has Dog	5749	-0.25 (-0.34, -0.16)	< 0.001*	-0.09 (-0.17, -0.02)	0.015*
Has Cat	5749	-0.11 (-0.18, -0.03)	0.007*	-0.07 (-0.13, 0.00)	0.054
Has other/miscellaneous pets	5749	-0.12 (-0.19, -0.04)	0.002*	-0.08 (-0.14, -0.02)	0.013*
**Total summary score**					
Has any Pet	5756	-0.58 (-0.79, -0.36)	< 0.001*	-0.29 (-0.47, -0.11)	0.002*
Has Dog	5756	-0.93 (-1.16, -0.69)	< 0.001*	-0.41 (-0.61, -0.21)	< 0.001*
Has Cat	5756	-0.24 (-0.45, -0.03)	0.026*	-0.12 (-0.29, 0.05)	0.174
Has other/miscellaneous pets	5756	-0.49 (-0.68, -0.29)	< 0.001*	-0.32 (-0.48, -0.15)	< 0.001*
**KS2**					
**Subject**	*N*	B (95% *CI*)	*p*	B (95% *CI*)	*p*
**English (score)**					
Has any Pet	2035	-0.08 (-1.56, 1.40)	0.915	-0.20 (-1.56, 1.15)	0.770
Has Dog	2037	-2.23 (-3.61, -0.86)	0.001*	-1.75 (-3.03, -0.47)	0.007*
Has Cat	2037	-1.17 (-2.45, 0.11)	0.072	-1.02 (-2.18, 0.15)	0.087
Has other/miscellaneous pets	2037	0.35 (-0.86, 1.56)	0.565	0.67 (-0.44, 1.79)	0.237
**Maths (score)**					
Has any Pet	2028	-2.48 (-4.41, -0.54)	0.012*	-1.34 (-3.13, 0.45)	0.142
Has Dog	2029	-2.95 (-4.75, -1.14)	0.001*	-2.27 (-3.95, -0.58)	0.009*
Has Cat	2029	-2.39 (-4.07, -0.71)	0.005*	-1.91 (-3.44, -0.37)	0.015*
Has other/miscellaneous pets	2029	-0.38 (-1.98, 1.21)	0.640	-0.02 (-1.48, 1.47)	0.998
**Science (score)**					
Has any Pet	2032	-0.95 –(2.00, 0.10)	0.077	-0.36 (-1.34, 0.62)	0.467
Has Dog	2034	-1.55 (-2.54, -0.58)	0.002*	-1.04 (-1.96, -0.11)	0.028*
Has Cat	2034	-1.48 (-2.39, -0.57)	0.001*	-1.20 (-2.04, -0.37)	0.005*
Has other/miscellaneous pets	2034	0.08 (-0.78, 0.95)	0.850	0.36 (-0.45, 1.16)	0.387
**Total summary score**					
Has any Pet	407	0.47 (-2.37, 3.31)	0.743	-0.40 (-3.06, 2.26)	0.768
Has Dog	407	-2.36 (-5.05, 0.32)	0.084	-1.63 (-4.19, 0.94)	0.215
Has Cat	407	-1.54 (-4.08, 0.99)	0.231	-0.46 (-2.76, 1.84)	0.698
Has other/miscellaneous pets	407	1.98 (-0.38, 4.34)	0.100	0.61 (-1.59, 2.82)	0.586
**GCSE**		OR (95% *CI*)		OR (95% *CI*)	
**Subject**					
English (A/A* versus B or lower)					
Has any Pet	1990	0.85 (0.71, 1.02)	0.081	0.84 (0.67, 1.05)	0.131
Has Dog	1993	0.63 (0.52, 0.75)	< 0.001*	0.75 (0.59, 0.94)	0.014*
Has Cat	1993	1.03 (0.87, 1.23)	0.716	0.88 (0.071, 1.09)	0.256
Has other/miscellaneous pets	1994	0.78 (0.66, 0.93)	0.004*	0.87 (0.71, 1.08)	0.203
Maths (A/A* versus B or lower)					
Has any Pet	1919	0.76 (0.63, 0.91)	0.003*	0.82 (0.66, 1.03)	0.089
Has Dog	1922	0.57 (0.48, 0.69)	< 0.001*	0.67 (0.54, 0.85)	0.001*
Has Cat	1922	0.85 (0.72, 1.02)	0.081	0.79 (0.63, 0.98)	0.035*
Has other/miscellaneous pets	1923	0.92 (0.77, 1.08)	0.305	1.03 (0.84, 1.27)	0.770
Biological Sciences (A/A* versus B or lower)					
Has any Pet	490	0.65 (0.45, 0.95)	0.024*	0.63 (0.39, 0.99)	0.048*
Has Dog	490	0.73 (0.52, 1.04)	0.084	0.76 (0.48, 1.20)	0.237
Has Cat	490	0.88 (0.63, 1.25)	0.485	0.93 (0.59, 1.45)	0.750
Has other/miscellaneous pets	490	0.60 (0.43, 0.84)	0.003*	0.61 (0.40, 0.92)	0.019*
Chemistry (A/A* versus B or lower)					
Has any Pet	478	0.56 (0.38, 0.82)	0.003*	0.46 (0.29, 0.73)	0.001*
Has Dog	478	0.65 (0.45, 0.92)	0.016*	0.63 (0.40, 0.99)	0.049*
Has Cat	478	0.83 (0.59, 1.17)	0.287	0.79 (0.52, 1.23)	0.305
Has other/miscellaneous pets	478	0.63 (0.45, 0.88)	0.007*	0.56 (0.37, 0.84)	0.005*
Physics (A/A* versus B or lower)					
Has any Pet	475	0.76 (0.59, 0.97)	0.027*	0.86 (0.64, 1.16)	0.310
Has Dog	476	0.63 (0.49, 0.80)	< 0.001*	0.83 (0.61, 1.12)	0.228
Has Cat	476	0.82 (0.65, 1.05)	0.114	0.76 (0.57, 1.03)	0.075
Has other/miscellaneous pets	476	0.85 (0.68, 1.06)	0.153	1.01 (0.76, 1.34)	0.935
Achieved 5 GCSEs A*-C					
Has any Pet	2010	0.89 (0.72, 1.09)	0.249	0.94 (0.73, 1.21)	0.634
Has Dog	2013	0.65 (0.54, 0.79)	< 0.001*	0.76 (0.60, 0.96)	0.023*
Has Cat	2013	1.06 (0.88, 1.29)	0.527	1.02 (0.80, 1.29)	0.873
Has other/miscellaneous pets	2014	0.89 (0.75, 1.07)	0.224	0.97 (0.78, 1.22)	0.818

*P < .05

Analyses were adjusted for: sex, maternal depression at ages 6, 8 and 11 years, maternal anxiety at ages 6 and 11 years, overcrowding at 7 and 10 years, house type at ages 7 and 10 years, highest parental social class (antenatal period), maternal education (antenatal period), maternal age at delivery (antenatal period), home ownership status (antenatal period), family income (antenatal period) and car ownership (antenatal period), school identifier, school type, birthweight, developmental delay measured at child age 30 months, child temperament at 2 years, older children living in the house (antenatal period), stressful life events at almost 4 years, 9 and 11 years old, and mother-child bonding at age 3

Results did not differ when accounting for parental marital status, maternal smoking, financial difficulties, dog walking, and time spent watching TV, outdoors and doing homework therefore these variables were discarded from the final models

At KS2 (Age 7–11), effect sizes were slightly larger (range 1.04–2.27 points lower score). Cross-sectional models demonstrated that owning a dog at this stage was associated with a lower score in English (b=-1.75, 95% CI=-3.03- -0.47, *p* = .007), as well as lower in Maths (b=-2.27, 95%CI=-3.95- -0.58, *p* = .009) and Science (b=-1.04, 95% CI=-1.96- − 0.011, *p* = .028) (Table 5). Owning cats was also associated with a poorer attainment in Maths (b=-1.91, 95% CI=-3.44- -0.37, *p* = .015) and Science (b=-1.20, 95% CI=-2.04- -0.37), *p* = .005) (Table 5).

At General Certificate of Secondary Education (GCSE – age 15–16), all pet types were associated in cross-sectional models with poorer attainment of an A/A* compared to B or lower, in a variety of subjects (Table 5). Specifically, owning any pet was associated with a lower likelihood of achieving top grades in Biological sciences (OR = 0.63, 95% CI = 0.39–0.99, *p* = .048), and Chemistry (OR = 0.46, 95% CI = 0.29–0.73, *p* = .001). Owning a dog was associated with a lower likelihood of achieving top grades in English (OR = 0.75, 95% CI = 0.59–0.94, *p* = .014), Maths (OR = 0.67, 95% CI = 0.54–0.85, p= ·001), Chemistry (OR = 0.63, 95% CI = 0.40–0.99, *p* = .049) and a lower likelihood of achieving five GCSEs A*-C (OR = 0.76, 95% CI = 0.60–0.96, *p* = .023). Owning a cat was associated with a lower likelihood of achieving top grades in Maths (OR = 0.79, 95% CI = 0.63–0.98, *p* = .035). Owning other pets was associated with a lower likelihood of achieving top grades in Biological Science (OR = 0.61, 95% CI = 0.40–0.92, *p* = .019), and Chemistry (OR = 0.56, 95% CI = 0.37–0.84, p= ·0.005).

When longitudinally exploring pet ownership history in relation to GCSE attainment, there was some evidence of an association (Additional file Supplementary Table 12). Specifically, there was weak evidence of an association between always owning pets up to 15 years and a lower likelihood of attainment of top grades in English (OR = 0.79, 95% CI = 0.63–0.99, *p* = .037), and strong evidence of an association with Maths OR = 0.67, 95% CI = 0.53–0.85, *p* = .001) compared to sometimes owning pets.

### Language Development

Owning a pet at age 5 was associated with a 1.01 (95% CI = 0.18–1.83, *p* = .017) higher Reynell Developmental Language Scale score at that age (Table 6). However, this association did not remain in a linear regression repeated measures design accounting for scores at both 2 and 5 years of age (b = 0.41, 95% CI=-2.04 -2.86, *p* = .743). Owning a pet at age 2 was associated with 0.18 points (95% CI = 0.04–0.3, *p* = .014) higher non-verbal communication score at that age (Table 6).

**Table 6:** Univariable and multivariable associations between pet ownership at ages 2 and 5, and language development scores

			Univariable		Multivariable (adjusted)	
Age		*N*	B (95% *CI*)	*p*	B (95% *CI*)	*p*
	**Reynell Developmental Language Scale**					
	Has any Pet					
2		713	-1.17 (-2.47, 0.13)	0.077	-0.04 (-1.13, 1.05)	0.843
5		393	0.39 (-0.35, 1.13)	0.299	1.01 (0.18, 1.83)	0.017*
	Has Dog					
2		712	-1.89 (-3.71, -0.07)	0.041*	-1.09 (-2.58, 0.39)	0.149
5		393	-0.24 (-1.25, 0.77)	0.646	0.93 (-0.19, 2.04)	0.103
	Has Cat					
2		712	-0.78 (-2.15, 0.60)	0.270	-0.15 (-1.29, 0.99)	0.800
5		393	0.27 (-0.53, 1.09)	0.502	-0.24 (-1.10, 0.63)	0.595
	Has other/miscellaneous pets					
2		712	-2.23 (-6.05, 1.58)	0.251	-2.76 (-6.04, 0.52)	0.099
5		393	0.65 (-1.33, 2.63)	0.520	0.87 (-1.13, 2.88)	0.393
2	**Total Communication Score (MacArthur)**					
	Has any Pet	6112	1.31 (-1.33, 3.95)	0.330	0.27 (-1.71, 2.25)	0.787
	Has Dog	6105	-1.01 (-4.43, 2.42)	0.565	-1.27 (-3.77, 1.24)	0.321
	Has Cat	6107	-0.89 (-3.77, 2.00)	0.547	-1.56 (-3.72, 0.59)	0.154
	Has other/miscellaneous pets	6108	-2.76 (-10.81, 5.29)	0.502	4.27 (-1.63, 10.18)	0.156
2	**Vocabulary Score (MacArthur)**					
	Has any Pet	6176	0.99 (-1.29, 3.28)	0.395	-0.02 (-1.80, 1.77)	0.985
	Has Dog	6169	-0.79 (-3.75, 2.17)	0.602	-1.13 (0.33, -3.39)	0.326
	Has Cat	6171	-0.72 (-3.22, 1.78)	0.572	-1.53 (-3.47, 0.42)	0.124
	Has other/miscellaneous pets	6172	-2.09 (-9.07, 4.88)	0.556	3.78 (-1.55, 9.13)	0.164
2	**Non-Verbal Communication Score (MacArthur)**					
	Has any Pet	6150	0.22 (0.05, 0.39)	0.014*	0.18 (0.04, 0.32)	0.014*
	Has Dog	6143	0.08 (-0.14, 0.31)	0.469	0.06 (-0.12, 0.24)	0.524
	Has Cat	6145	0.02 (-0.17, 0.21)	0.823	0.05 (-0.10, 0.21)	0.517
	Has other/miscellaneous pets	6146	-0.09 (-0.62, 0.44)	0.731	0.18 (-0.25, 0.60)	0.420
2	**Social Development Score (MacArthur)**					
	Has any Pet	6158	0.06 (-0.24, 0.36)	0.687	0.13 (-0.09, 0.36)	0.270
	Has Dog	6151	-0.41 (-0.79, -0.03)	0.035*	-0.21 (-0.49, 0.08)	0.159
	Has Cat	6153	-0.03 (-0.36, 0.29)	0.852	-0.03 (-0.27, 0.22)	0.829
	Has other/miscellaneous pets	6154	0.25 (-0.66, 1.16)	0.587	0.67 (-0.01, 1.35)	0.053

*P < .05

Analyses were adjusted for: sex, ethnicity, maternal depression at almost 2 and 4 years, maternal anxiety at ages almost 2 and 4 years, overcrowding at 2 years, house type at ages 2 and 3 years, highest parental social class, maternal education, maternal age at delivery, home ownership status, family income and car ownership, birthweight, child has twin, child attended day care at 15 months, and 4 years, number of languages spoken in the home, developmental delay at 18 months, child temperament, older children living in the house, stressful life events at almost 2 and 4 years old and mother-child bonding at age 3 years

Results did not differ when accounting for house type or dog walking (Number of times in typical week respondent walked or jogged with household dog(s)), therefore these variables were discarded from the final model

## Discussion

Pet ownership was positively associated with aspects of social development including language development, non-verbal communication and prosocial behaviour. However, contrary to popular belief, pet ownership did not appear to have any considerable beneficial association with emotional, cognitive or educational development of children. In fact, pet ownership was associated with lower educational attainment across a number of assessments in Key stage 1 and 2 and a range of subjects including Maths, English, and Sciences. Furthermore, at age 8, children who owned any pets had lower odds of high self-esteem (scholastic competence). The present findings are largely inconsistent with our hypotheses and previous research in the field - the majority being qualitative research- which tends to find positive effects of pets on child development, particularly in emotional health [11].

### Emotional health

Our findings are at odds with a common belief that companion animals benefit the emotional health of children and young people, formulated on the basis of results from other cross-sectional and qualitative studies previously reviewed [11]. The potential association we found between cat ownership and lower self-perceived scholastic competence was unexpected and could be due to residual confounding. It is also unclear why ‘any’ and ‘other’ pet ownership was associated with a higher odds of social anxiety symptoms at age 7. A prospective study of children aged 8–12 years [72] also found pet ownership to be partly detrimental to the level of children’s social interaction; attachment to pets at the 12 month follow-up was associated with both increases in the amount of time spent alone and decreases in children’s time spent with family and friends, which in turn may influence social anxiety.

Our mostly null results regarding emotional health support those of a recent study using a longitudinal design [46]; this propensity-score-weighted population-based study established that any benefits of owning companion animals on psychological health in children and adolescents were largely explained by confounding factors. Their results also suggested that factors such as socio-economic status are more important predictors of emotional health in children and young people than animals. The buffering hypothesis [73] may also help explain our mostly null findings; social support and emotional benefits of companion animals may only come into play for individuals experiencing adverse or stressful events. Population-based studies are not well suited to detect such effects [46]. Furthermore, it is important to recognize that, at least in some instances, animal caregiving may elicit negative emotions related to pet care, health, and separations [27, 74] and strong attachment to animals has been associated with emotional distress and depressive symptoms [27, 75].

### Behavioural and cognitive development

Owning ‘other/miscellaneous’ pets at age 3 was associated with a lower likelihood of experiencing prosocial difficulties, and dog ownership at age 3 with increased score for prosocial behaviours (kind to others and animals, considerate of other’s feelings, shares toys). Other studies have found similar effects in similarly-aged children [76, 77]. Therefore companion animals may be hypothesized to indirectly increase young children’s prosocial behaviour through acting as a point of conversation between adults, peers and children [77], or by adults teaching children how to correctly care for animals [78].

However, the ownership of cats at age 3 was associated with higher odds of hyperactivity but not at age 11, and pet/dog/cat ownership also showed some associations with conduct problems at ages 3 and 11 years. Similarly, in other research children owning pets have been found to be more likely to have ADHD [46], hyperactivity/inattention and also conduct problems [79]. This finding has been previously attributed to the exposure of increased microbial contact such as mould and dampness caused by indoor pets potentially affecting cognitive function, however, we were able to control for housing defects within ALSPAC and our associations did not attenuate so this explanation seems unlikely. Addressing an identified research gap on companion animals and cognitive development of children, including executive function [11], our study also found no evidence of positive associations here, potentially supporting the behavioural findings. Surprisingly, dog ownership was associated with a poorer ability in attentional switching only at age 11. Attentional switching or ‘cognitive flexibility’ is arguably the most complex executive function as it both requires and builds on inhibition and working memory [80]. Studies on executive attention show a developmental transition from perceptual to executive attention between the ages of 9 and 12 years [81], and the Dual Task used may not be the most methodologically accurate way to capture the complexity of attentional switching in this age group [82].

An alternative explanation for our findings regarding behavioural difficulties and cognitive development is reverse causation. There is evidence that families may acquire dogs to support children with developmental delays or disabilities (or at least, symptoms of poorer performance or disability) [83]. Therefore one might expect that higher incidence of behavioural difficulties, or poorer performance in attentional switching as seen in ADHD [84] and often Autism Spectrum Disorder (ASD) [85], would be found in animal owners.

Findings did not support previous research where animal presence during task completion has positive effects on memory and attention [37, 38, 86]. This is likely due to the closer proximity and direct involvement of the animal in these experimental tests, and potentially to these animals not being participants’ pets. The dopamine release from an interaction with a dog [87], which is known to enhance concentration and attention [88], may be too distal to ALSPAC children who completed the task in CiF clinics whilst the pets were at home.

### Educational development

This was the first known study to assess whether pet ownership in childhood and adolescence is associated with educational attainment. Pet ownership was consistently associated with poorer educational attainment at KS1, KS2 and GCSE in a variety of subjects, and findings were generally consistent across pet types, although effect sizes detected were sometimes very small, ranging 0.07–2.27 points lower for SATs and 0.79 − 0.46 lower odds (equivalent to 1.3–2.4 times more likely) of achieving high GCSE grades. No evidence was found in ALSPAC supporting previous research that pet owners may be better at biological subjects [89, 90].

It is possible that the association between pet ownership and poorer educational attainment is attributable to unknown confounding factors. To explore this, we further adjusted using multivariable modelling for additional hypothetical variables including parental marital status, maternal smoking, financial difficulties, dog walking, time spent watching TV, time spent outdoors and time spent doing homework, with no change in effect. Again, it may be hypothesized that parents may acquire dogs to support children with poorer educational attainment.

### Language development

This was also the first study to examine potential associations between pet ownership and language development outcomes in childhood. Owning a pet was associated with a higher comprehension score at age 5 (RDLS) of about 1 point on the scale. In contrast to 2-year-olds who are still in the stages of word learning, 5-year-olds can communicate with their animals as social partners and can talk to them more. Five-year-olds may also utilise multiple cues pets may give better, and they may employ social-pragmatic factors and global attentional mechanisms [91] to interpret pet behaviour and talk to their animals. This area requires further investigation.

Interestingly, owning a pet at age 2 was associated with slightly higher non-verbal communication scores. Non-verbal communication includes eye gaze, vocalizations, and prelinguistic gestures. It is possible that young children whose families own animals are better practised in such body language and communication, to enable them to interact with the animal, or with parents/siblings to communicate about the animal as an additional and highly interesting object of interest. Interaction with animals may enhance social cues such as eye gaze, pointing, and speaker intention, needed for language development through usage-based language acquisition theory [92]. Again, this will need further systematic investigation.

Our findings support previous suggestions that owning animals may facilitate language acquisition, with the pet acting as a recipient of conversation, an additional subject of conversation, to stimulate communication, and potentially build vocabulary [77]. The presence of dogs has also been shown to influence reading ability in school age children [93]. Further research is needed in particular to understand whether companion animals can enhance other aspects of language e.g., semantics, pragmatics or syntactic development.

### Limitations

The ALSPAC dataset has many strengths as it is a large community-based cohort with availability of data on a wide range of developmental outcomes. It also has the ability to adjust for confounding factors (which pertinently attenuated effects in some models). Consistent findings to ours from one other well-designed longitudinal study [46] indicate that more research is needed, including well designed longitudinal studies and randomised controlled trials.

However, some limitations should be considered when interpreting our findings. First, our mostly small effect sizes observed may not relate to clinically significant differences for individuals. We did examine numerous outcomes so some of our findings could be due to statistical chance – for this reason and as recommended we have not used statistical cut offs but presented the full *P* Value and 95% confidence intervals so the reader can make their own interpretation [70]. We also emphasise patterns in our interpretation of findings rather than relying on single associations. Further, ALSPAC did not measure the child’s relationship with or ‘attachment’ to their companion animals directly, for example using a validated measure, and it is precisely this relationship that may be most salient in conferring psychological benefits, rather than the presence of companion animals as such. However, analyses of the pet-interaction variable which may have acted as a substitute for attachment presented no evidence of associations. We also explored history of pet ownership, i.e., a longitudinal and cumulative perspective, which again presented us with no evidence of associations. In addition, as the emotional, behavioural and cognitive development of young people is a dynamic process, variables are not always fully independent, but can influence each other. There are complexities when examining such health outcomes and it is difficult to tease apart associations, for example, poor self-esteem may lead to depression and vice versa. Likewise, reverse causality may be occurring; if families of children with mental, behavioural or cognitive health problems are more likely to get a pet to alleviate symptoms and support parents and children, then at a cross-sectional examination one could expect worse, not better, mental health, behaviour and cognition in children with companion animals – which fits our results to some extent. Future research should investigate causal pathways in more detail for each type of outcome. We also did not examine interactions between variables and these should be considered in future more detailed research into outcomes where suggested associations are present, for example whether results differ by sex or socioeconomic status. Finally for consideration, due to the nature of the cohort recruitment and attrition, the sample involves mainly white, more affluent children in the UK, and findings may not generalise outside these contexts.

## Conclusions

In conclusion, the present study found evidence of improved language and prosocial development in children who owned companion animals. However, it also found isolated and small associations between pet ownership and poorer self-esteem, higher social anxiety, more hyperactivity and conduct difficulties, and poorer cognitive ability in dual attention. Further, consistently poorer educational attainment was seen across many types of pet ownership. Our data evidences that companion animals may have a sociability function in children that may echo that seen in adults [25, 94].

Whilst our study provides novel evidence and demonstrates the importance of using large, well-designed longitudinal studies and controlling for key confounders, it also illustrates the complexity and importance of studying the impact of companion animal ownership on childhood development. Future cohort studies should collect detailed information on children’s relationships with their companion animals, including validated measures of pet attachment and the nature of interactions with the animal. Longitudinal investigation using repeated measures, and potentially randomised experimental conditions, if possible, are also required in order to determine cause and effect, and to determine clinical significance of any differences.

## Electronic supplementary material

Below is the link to the electronic supplementary material.

Supplementary Material 1

## Data Availability

Requests to access ALSPAC data can be made via https://www.bristol.ac.uk/alspac/researchers/access/.

## Abbreviations

ALSPAC: Avon Longitudinal Study of Parents and Children
CiF: Children in Focus Clinic
DAWBA: Development And Wellbeing Assessment
GCSE: General Certificate Secondary Education
HSPP: Harter Self-Perception Profile
KS1: Key Stage 1
KS2: Key Stage 2
MCDI: MacArthur Communication Development Inventories
MFQ: Mood and Feelings Questionnaire
RDLA: Reynell Development Language Scale
RRS: Revised Rutter Scale
SAT: Standard Assessment Test
SDQ: Strengths and Difficulties Questionnaire
TEACh: Tests of Everyday Attention for Children
