# Supplementary material for: Companion animals and child development outcomes: longitudinal and cross-sectional analysis of a UK birth cohort study
Source: BMC Pediatr. 2024 Sep 13;24:578. doi: 10.1186/s12887-024-05049-7 (PMC11395694; doi:10.1186/s12887-024-05049-7)
Supplement: Supplementary file 1 — Supplementary Material 1 [file 12887_2024_5049_MOESM1_ESM.docx]

Additional File

# **Companion Animals and Child Development Outcomes: Longitudinal and Cross-sectional Analysis of a UK Birth Cohort Study**

Rebecca Purewal, Robert Christley, Katarzyna Kordas^,^ Carol Joinson^,^ Kerstin Meints^,^ Nancy Gee and Carri Westgarth

ADDITIONAL METHODS

Emotional Health Outcomes

*Self-esteem*

Children aged 8.5 years (on average) completed a shortened version of Harter’s Self Perception Profile for Children (SPPC) (Harter, 1985) at a face-to-face CiF clinic. Low self-esteem in scholastic competence was defined as a score ≤14; low self-esteem in global self-worth as a score ≤16 (Supplementary table 3).

**Supplementary figure 1.** Flow chart depicting sample sizes and derivation for self-esteem at age eight

Multiple Imputation (N= 3938)

Global self-worth

Multiple Imputation (N= 3951)

Have pet ownership information (N= 7651)

Have pet ownership information (N= 7651)

Complete case (N= 3044)

Complete case (N= 3056)

SPPC response (N= 6935)

SPPC response (N=6946)

Starting sample (N= 13,954)

Scholastic competence

*Anxiety*

The Development and Well-Being Assessment (DAWBA) (Goodman et al., 2000) was used to measure symptoms of separation anxiety, social anxiety and generalized anxiety disorder at 7, 10 (parent-completed) and 13 (child-completed) years, and depression at 7 years (parent-completed). Due to the low rate of DSM-IV disorders in ALSPAC, analyses were conducted using a set of dichotomous outcome variables derived from the lists of symptoms in the DAWBA relating to each emotional health outcome. These binary scores were derived as detailed in Supplementary table 3.

**Supplementary figure 2**. Flow chart depicting sample sizes and derivation for separation anxiety

Starting sample (N= 13,954)

Age 13

Age 7

Age 10

Pet ownership information (N=3050)

Pet ownership information (N=7800)

Pet ownership information (N=8331)

DAWBA response (N= 6464)

DAWBA response (N= 7282)

DAWBA response (N= 7391)

Multiple Imputation (N= 2390)

Multiple Imputation (N= 6638)

Complete case (N= 1416)

Complete case (N= 3671)

Complete case (N= 3266)

Multiple Imputation (N= 6375)

**Supplementary figure 3.**  Flow chart depicting sample sizes and derivation for social anxiety

Starting sample (N= 13,954)

Age 13

Age 10

Age 7

Pet ownership information (N=7800)

Pet ownership information (N=8331)

DAWBA response (N= 7051)

Pet ownership information (N=3050)

DAWBA response (N= 7678)

DAWBA response (N= 8052)

Multiple Imputation (N= 2408)

Multiple Imputation (N= 6714)

Multiple Imputation (N= 7208)

Complete case (N= 1543)

Complete case (N= 3423)

Complete case (N= 3963)

**Supplementary figure 4.** Flow chart depicting sample sizes and derivation for generalised anxiety disorder

Starting sample (N= 13,954)

Age 13

Age 10

Age 7

Pet ownership information (N=7800)

Pet ownership information (N=8331)

DAWBA response (N= 2849)

Pet ownership information (N=3050)

DAWBA response (N= 3298)

DAWBA response (N= 8101)

Multiple Imputation (N= 2890)

Multiple Imputation (N= 7244)

Complete case (N= 3981)

Multiple Imputation (N= 1126)

Complete case (N= 1559)

Complete case (N= 679)

*Depression*

Depressive symptoms at age 10 and 13 years were assessed using the short Mood and Feelings Questionnaire (MFQ) completed by children at Children in Focus (CiF) research clinics (Angold et al., 1995; Messer et al., 1995). The MFQ consists of 13 items enquiring about the occurrence of depressive symptoms over the past 2 weeks. The cut-off point of 11 was used to indicate high levels of depressive symptoms (Additional Table 3).

**Supplementary figure 5.** Flow chart depicting sample sizes and derivation for depression

Starting sample (N= 13,954)

Age 13

Age 10

Age 7

MFQ response (N= 6019)

Have pet ownership information (N=3050)

Have pet ownership information (N=7800)

Have pet ownership information (N=8331)

MFQ response (N= 7363)

DAWBA response (N= 8039)

Multiple Imputation (N= 6054)

Multiple Imputation (N= 2332)

Complete case (N= 1366)

Complete case (N= 3069)

Multiple Imputation (N= 7194)

Complete case (N= 3952)

Behavioural Outcomes

*Revised Rutter Parent Scale for Preschool Children*

The Revised Rutter Parent Scale for Preschool Children (RRS) (Rutter et al., 1970) was administered to parents and caregivers to measure child behaviour at age 3 (42 months). Subscales consisted of four different behavioural aspects; emotional difficulties, conduct difficulties, hyperactivity-inattention difficulties and prosocial behaviour domains (high score indicated more problems). A total behavioural difficulties score was calculated from the sum of the subscales (excluding prosocial behaviour as advised by user manual). Binary scores were derived; as no clinical cut-off score exists for the RRS, cut-offs were calculated using standardized z scores, taking the highest (or lowest) tertile of the RRS subscale.

*Strengths and Difficulties Questionnaire*

In older children, behavioural problems were assessed using the Strengths and Difficulties Questionnaire (SDQ), which was completed by the caregiver when the child reached 11 years (140 months). Similar to the RRS, subscales consisted of emotional difficulties, conduct difficulties, hyperactivity-inattention difficulties, prosocial behaviour, and, in addition, peer relationship difficulties. A total behavioural difficulties score was calculated from summing the scores of the subscales (excluding prosocial behaviour). Binary scores were derived using clinical cut off scores for subscales (emotional difficulties=4, conduct difficulties=4, hyperactivity-inattention difficulties=6, prosocial behaviour=5, and peer relationship difficulties=3, total behavioural difficulties=14).

**Supplementary figure 6.** Flow chart depicting sample sizes and derivation for behaviour

Starting sample (N= 13,954)

SDQ response (N= 9526)

Complete case (N= 2058)

Age 11

Age 3

RRS response (N= 10,018)

Have pet ownership information (N= 3063)

Have pet ownership information (N= 9576)

Multiple Imputation (N= 8963)

Multiple Imputation (N= 2643)

Complete case (N= 7173)

Cognitive Outcomes

Children were invited to, and underwent testing for all cognitive outcomes at CiF clinics at ages 8, 10 and 11.

*Attention*

The outcome measures for attentional functioning were derived from the Test of Everyday Attention for Children (TEACh) (Manly et al., 1998). Selective attention, attentional switching and attentional control were measured.

1. Selective Attention (Sky Search Task)

The Sky Search task was completed to assess selective attention at ages 8 and 11 years. As recommend in the manual, motor processing reaction time was subtracted from the ability score to provide a final score of selective attention. A higher score indicates a more impaired selective attention.

2. Attentional Switching (Sky Search Dual Task)

The Sky Search Dual task followed the same procedure as the Sky Search task but with the addition of simultaneously presented auditory stimuli. The final score Dual Task score was calculated by taking the Sky Search Task’s score (prior to adjusting for motor performance), from the score created from the Dual Task Score itself. A higher score indicated a more impaired ability in attentional switching.

3. Attentional Control (Same and Opposite World task)

Attentional control was measured at ages 8 and 11 using the Same and Opposite Worlds tasks. The mean time taken to complete the same world trials was taken as a measure of verbal processing. The mean time on the opposite world trials was taken as the measure of attentional control: higher reaction times indicate more impaired ability.

*Impulsivity/inhibitory control*

Impulsivity was measured at age 10 years using the Stop-Signal task at a research clinic. Performance on this task was measured as number of correct stop signal trials at 150 and 250ms delay before the mean reaction time.

*Working Memory*

The Digit Span Task to assess working memory was completed at age 10. A working memory span score was calculated as the number of correctly recalled sets weighted by the number of screens within each set.

**Supplementary figure 7.** Flow chart depicting sample sizes and derivation for cognition age 8

Starting sample (N= 13,954)

Dividing Attention

Working memory

Selective Attention

Attentional control

Counting span (N= 7197)

Have pet ownership information (N= 7651)

Dual task (N= 6552)

Have pet ownership information (N= 7651)

Have pet ownership information (N= 7651)

Have pet ownership information (N= 7561)

Same/opp worlds (N= 7207)

Sky search (N= 7181)

Multiple Imputation (N= 5720)

Complete case (N= 3748)

Multiple Imputation (N= 5749)

Complete case (N= 3429)

Complete case (N= 3779)

Multiple Imputation (N= 5228)

Multiple Imputation (N= 5745)

Complete case (N= 3765)

**Supplementary figure 8.** Flow chart depicting sample sizes and derivation for cognition at age 10

Starting sample (N= 13,954)

)

Impulsivity

Working memory

Stop signal (N= 7522)

Counting span (N= 7006)

Have pet ownership information (N= 7800)

Have pet ownership information (N= 7800)

Multiple Imputation (N= 5760)

Multiple Imputation (N= 5729)

Complete case (N= 3302)

Complete case (N= 3317)

**Supplementary figure 9.** Flow chart depicting sample sizes and derivation for cognition at age 11

Starting sample (N= 13,954)

Attentional control

Dividing Attention

Selective Attention

Same/opp world (N= 6796)

Have pet ownership information (N=3063)

Have pet ownership information (N=3063)

Have pet ownership information (N= 3063)

Multiple Imputation (N= 2448)

Dual task (N= 6543)

Multiple Imputation (N= 2357)

Sky search (N= 6552)

Complete case (N= 1536)

Complete case (N= 1640)

Multiple Imputation (N= 2522)

Complete case (N= 1595)

Educational Outcomes

*SAT (KS1 and KS2)*

In childhood, Standardised Assessment Test (SAT) results were used to measure educational attainment. ALSPAC obtained Key Stage 1 (KS1) (Reading, Writing and Mathematics) and Key Stage 2 (KS2) (English, Mathematics and Science) results from Local Education Authorities.

*GCSE (KS4)*

In adolescence, General Certificate of Secondary Education (GCSE) results were used to measure educational attainment, typically at age 16 years. Six outcomes relating to GCSE attainment were used in the analysis: English, Maths, Biological Sciences, Chemistry, Physics, and whether the child achieved five GCSE grades A*-C. GCSE subject scores were derived to examine whether the child achieved optimal grades (A* or A compared to B-G).

**Supplementary figure 10.** Flow chart depicting sample sizes and derivation for Education

Starting sample (N= 13,954)

KS1

GCSE

KS2

Have pet ownership information (N= 3034)

Have pet ownership information (N= 7800)

Have pet ownership information (N= 8331)

KS4 GCSE (N= 12,290)

Multiple Imputation (N= 2010)

KS2 SAT (N= 2249)

Multiple Imputation (N= 407)

KS1 SAT (N= 11,551)

Complete case (N= 1570)

Complete case (N= 306)

Multiple Imputation (N= 5756)

Complete case (N= 4166)

Language Development Outcomes

*Reynell Developmental Language Scales*

Reynell Developmental Language Scales (RDLS) (Reynell & Curwen, 1977) (RDLS Comprehension Scale) were used to measure language ability at ages 2 and 5 years in CiF clinics.

*Macarthur*

An ALSPAC adaptation of the MacArthur Toddler Communication questionnaire (MCDI) (Fenson et al., 1991) was administered to the mother/caregiver of children aged 2 years. The MCDI allows clinicians to assess early language, non-verbal, and social development in children. A vocabulary, non-verbal communication, social development and total communication score was assessed.

**Supplementary figure 11.** Flow chart depicting sample sizes and derivation for language development

Complete case (N= 4316)

Starting sample (N= 13,954)

Complete case (N= 298)

Age 2

Age 5

Have pet ownership information (N= 9706)

MacArthur (N= 10,861)

RDLS (N= 988)

Have pet ownership information (N= 9576)

Complete case (N= 511)

Multiple Imputation (N= 6112)

Multiple Imputation (N= 713)

Multiple Imputation (N= 393)

RDLS (N= 1127)

Confounding factors

Sex: Medical records at birth, male or female.

Ethnicity: Carer questionnaire at 140 months, White or Other.

Socioeconomic status: highest parental social class (Professional (highest), Managerial and technical, Skilled: non-manual, Skilled: manual, Partly skilled, Unskilled (lowest) (OPCS, 1991) classification), maternal education (CSE or no qualification (lowest), vocational, O level, A level, degree (highest)), grouped maternal age at delivery (<21, 21-30, >30), overcrowding (>5 people), house type (detached, semi-detached, end-terrace, terraced, flat/room in someone else’s house/other), financial difficulties (occurrence of major financial problems since pregnancy versus none), ownership of home (owned accommodation; privately rented; subsidized housing), and housing defects, family income and car access (these variables were mainly derived from questionnaires administered during the antenatal period).

Family factors: older children living with the child, whether the child has a twin, if the child attends day care, and parental marital status (ALSPAC maternally reported questionnaires (Joinson et al., 2016))

Maternal emotional health: maternal depression (Edinburgh Postnatal Depression Scale EPDS (Cox et al., 1987) dichotomized at a cut-off of 13), and maternal anxiety (Crown-Crisp Experiential Index CCI (Birtchnell et al., 1988) dichotomized at a cut-off of 9).

Child factors: Where appropriate, models have been adjusted for developmental delay (Denver development scale (Frankenburg & Dodds, 1967)), child temperament (Toddler Temperament Scale (Carey & McDevitt, 1978)), and stressful live events score (ALSPAC maternally reported questionnaires (Joinson et al., 2016)).

DAG for deciding appropriate confounding variables are provided in Supplementary figures 12-14

Legend for figures 12-14


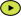
 exposure


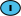
 outcome


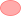
 ancestor of exposure *and* outcome


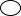
 adjusted variable


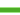
 causal path


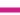
 biasing path

**Supplementary figure 12.** Causal model for PO and emotional health outcomes


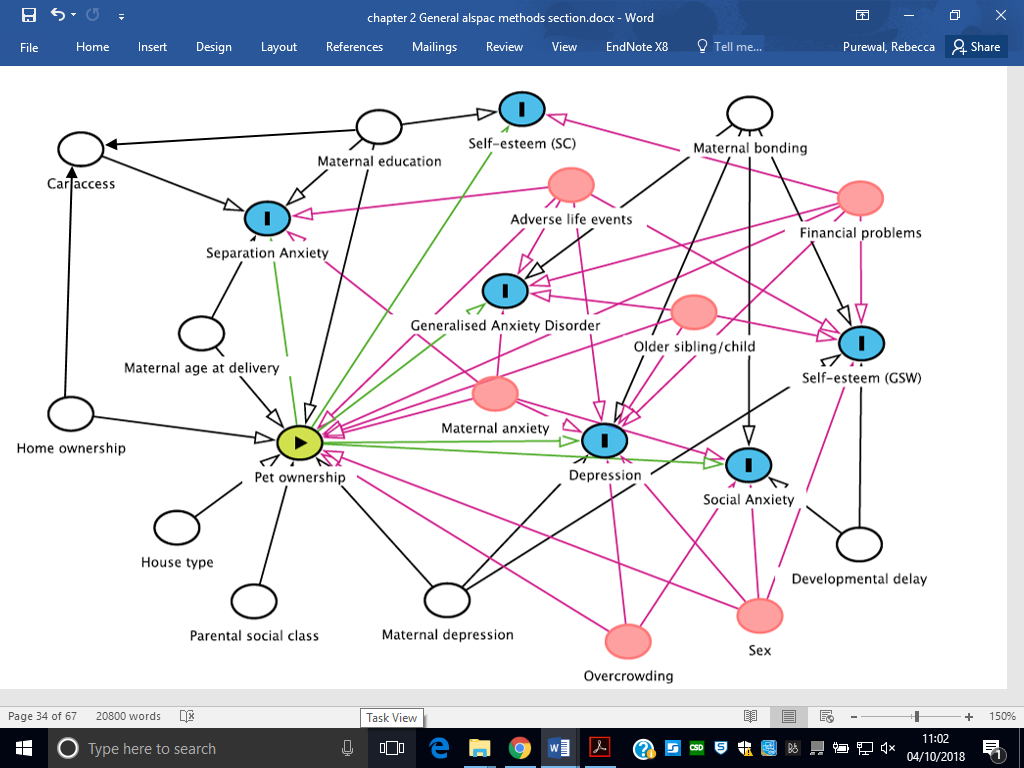


**Supplementary figure 13.** Causal model for PO, cognitive and educational outcomes


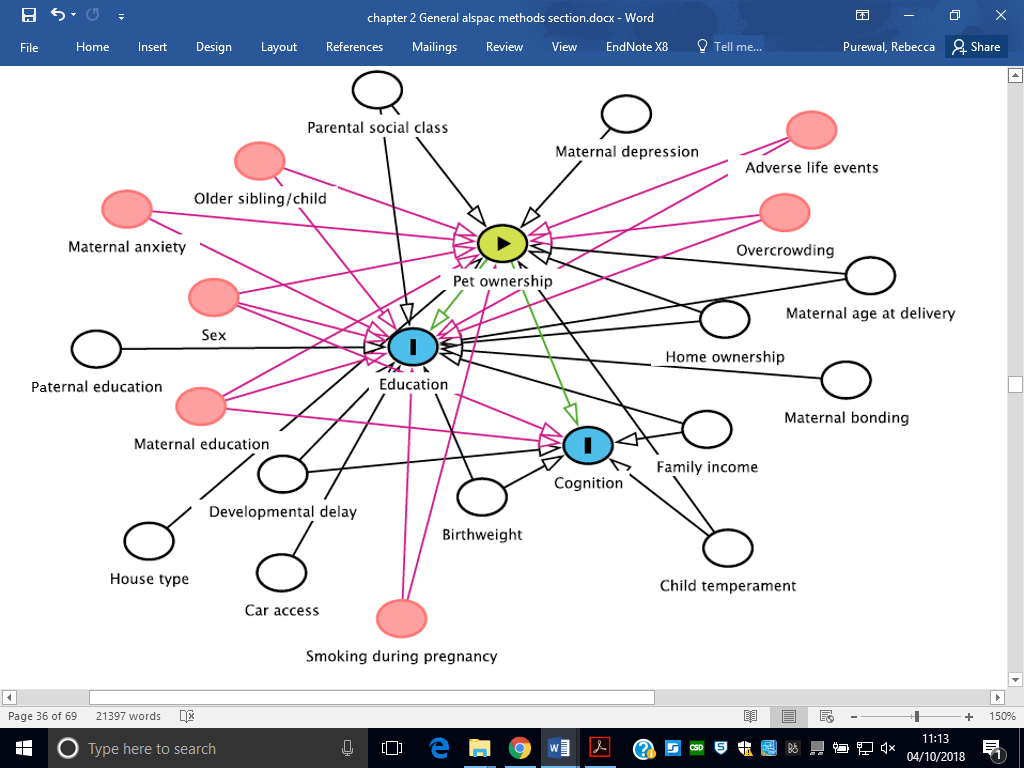


**Supplementary figure 14.** Causal model for PO, language and behavioral outcomes


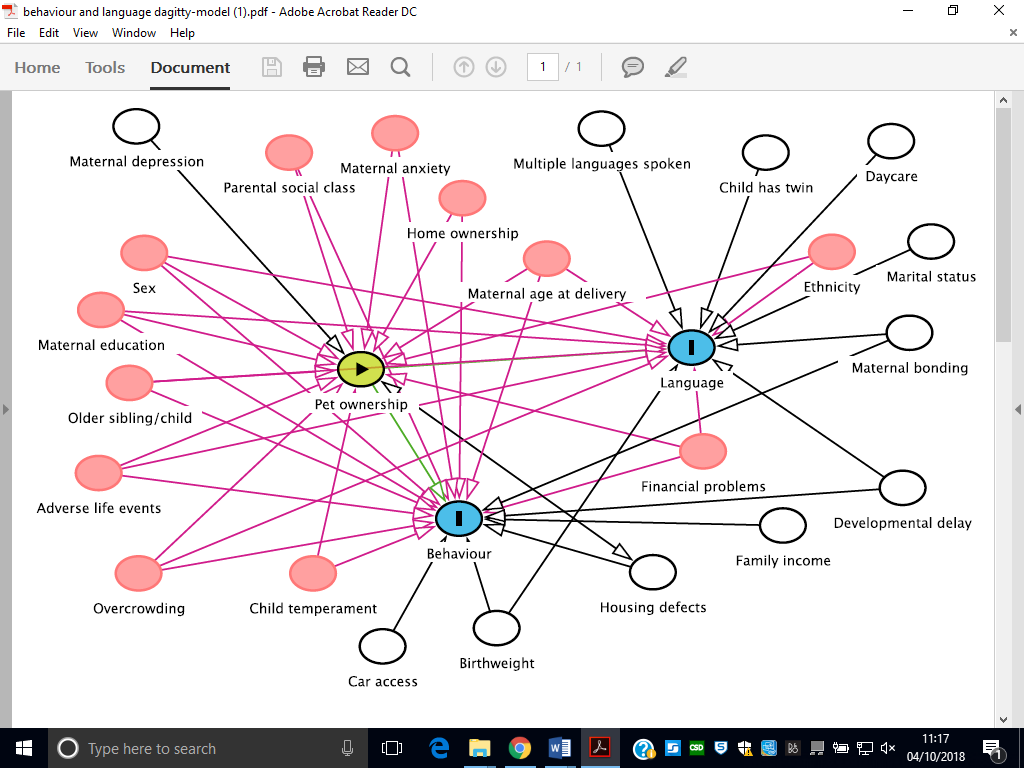


Missing data for each confounder is presented in Supplementary Table 1.

**Supplementary Table 1.** Number (%) of participants with missing data on each confounding variable.

| Outcome/Confounder | Original dataset (at gestation, *N* = 15455)  *N* (%) | PO data (age 2, *N* = 9700)  *N* (%) | PO data (age 3, *N* = 9576 )  *N* (%) | PO data (age 7, *N* = 8325)  *N* (%) | PO data (age 8, *N* = 7646)  *N* (%) | PO data (age 10, *N* = 7794)  *N* (%) | PO data (age 11, *N* = 3058)  *N* (%) | PO data (age 13, *N* = 3044)  *N* (%) | PO data (age 15, *N* = 3034 )  *N* (%) |
| --- | --- | --- | --- | --- | --- | --- | --- | --- | --- |
| Sex | 7 (0) | 1 (0) | 1 (0) | 0 (0) | 0 (0) | 0 (0) | 1 (0) | 1 (0) | 1 (0) |
| Ethnicity | 5 (0) | -- | -- | -- | -- | -- | -- | -- | -- |
| Birthweight | 7 (0) | 65 (1) | -- | -- | -- | -- | 133 (6) | -- | -- |
| Child has Twin | 3978 (26) | 3509 (61) | 3509 (61) | -- | -- | -- | -- | -- | -- |
| Maternal depression |  |  |  |  |  |  |  |  |  |
| Age 2 | 5856 (38) | 72 (1) | -- | -- | -- | -- | -- | -- | -- |
| Age 4 | 5856 (38) | -- | 142 (3) | -- | -- | -- | -- | -- | -- |
| Age 6 | 6839 (44) | -- | -- | 716 (9) | -- | -- | -- | -- | -- |
| Age 8 | 7543 (49) | -- | -- | -- | 49 (1) | 1132 (15) | -- | -- | -- |
| Age 11 | 7646 (50) | -- | -- | -- | -- | -- | 309 (13) | 395 (12) | 391 (25) |
| Maternal anxiety |  |  |  |  |  |  |  |  |  |
| Age 2 | 5878 (38) | 92 (2) | -- | -- | -- | -- | -- | -- | -- |
| Age 4 | 5878 (38) | -- | 164 (4) | -- | -- | -- | -- | -- | -- |
| Age 6 | 6846 (44) | -- | -- | 721 (9) | 725 (9) | 894 (11) | -- | -- | -- |
| Age 11 | 7805 (51) | -- | -- | -- | -- | -- | 310 (13) | 397 (12) | -- |
| Highest parental social class | 3885 (25) | 444 (8) | 802 (17) | 606 (7) | 504 (7) | 578 (7) | 225 (9) | 296 (10) | 392 (25) |
| Maternal education | 2968 (19) | 165 (3) | 291 (6) | 232 (3) | 196 (3) | 234 (3) | 156 (7) | 195 (6) | 189 (12) |
| Older children living with child | 4325 (28) | 253 (5) | 453 (9) | 264 (3) | 353 (5) | 429 (6) | 213 (9) | 273 (9) | 267 (17) |
| Maternal age at delivery | 1363 (9) | 0 (0) | 0 (0) | 0 (0) | 0 (0) | 0 (0) | 98 | 126 (4) | 122 (8) |
| Overcrowding |  |  |  |  |  |  |  |  |  |
| Age 2 | 5301 (34.3) | 489 (9) | -- | -- | -- | -- | -- | -- | -- |
| Age 7 | 7070 (46) | -- | -- | 16 (0) | -- | -- | -- | -- | -- |
| Age 8 | 7782 (50) | -- | -- | -- | 7 (0) | -- | -- | -- | -- |
| Age 10 | 7692 (50) | -- | -- | -- | -- | 61(1) | 360 (14) | 453 (15) | 448 (28) |
| House type |  |  |  |  |  |  |  |  |  |
| Age 2 | 5126 (33) | 715 (15) | -- | -- | -- | -- | -- | -- | -- |
| Age 3 | 5783 (37) | -- | 715 (14) | -- | -- | -- | -- | -- | -- |
| Age 7 | 7069 (46) | -- | -- | 43 (1) | 695 (9) | -- | -- | -- | -- |
| Age 10 | 7329 (48) | -- | -- | -- | -- | 38 (0) | -- | 352 (12) | 286 (22) |
| Financial difficulties | 4126 (27) | 278 (5) | 474 (9) | 376 (5) | 340 (4) | 399 (5) | 202 (8) | 269 (9) | 269 (18) |
| Family income | 6608 (42.8) | 478 (9) | 478 (9) | -- | -- | -- | 438 (18) | -- | 416 (19) |
| Ownership of home | 4212 (27) | 306 (6) | 306 (6) | 419 (5) | 373 (5) | 437 (6) | 209 (9) | 283 (9) | 277 (20) |
| Car access | 4199 (27) | 307 (6) | 307 (6) | 418 (5) | 378 (5) | 440 (6) | 209 (9) | 283 (9) | 276 (18) |
|  |  |  |  |  |  |  |  |  |  |
| Housing defects | 1731 (11) | 87 (2) | -- | -- | -- | -- | 125 (5) | -- | -- |
| Denver development scale | 5201 (34) | 338 (6) | 338 (6) | 559 (7) | 534 (7) | 638 (8) | 273 (11) | 355 (12) | 351 (23) |
| Child temperament | 5124 (33) | 382 (7) | -- | -- | -- | -- | 250 (10) | -- | -- |
| Stressful life events |  |  |  |  |  |  |  |  |  |
| Age 2 | 5050 (33) | 383 (7) | -- | -- | -- | -- | -- | -- | -- |
| Age 4 | 5855 (38) | -- | 537 (10) | 583 (7) | 577 (8) | -- | -- | -- | -- |
| Age 9 | 7415 (48) | -- | -- | -- | -- | 908 (12) | -- | -- | -- |
| Age 11 | 7792 (51) | -- | -- | -- | -- | -- | 309 | 396 (13) | 391 (25) |
| IQ | 8091 (52) | -- | -- | -- | 1777 (23) | -- | -- | -- | -- |
| School Identifier | -- | -- | -- | -- | -- | -- | -- | -- | 3188 (21) |
| School Type | -- | -- | -- | -- | -- | -- | -- | -- | 3155 (20) |
| Day care |  |  |  |  |  |  |  |  |  |
| Age 1 | 4502 (29) | 512 (9) | 552 (11) | -- | -- | -- | -- | -- | -- |
| Age 4 | 5892 (38) | 652 (12) | 652 (12) | -- | -- | -- | -- | -- | -- |
| Number of languages spoken | -- | 5314 (34) | -- | -- | -- | -- | -- | -- | -- |
| Parenting |  |  |  |  |  |  |  |  |  |
| Maternal enjoyment | 5876 (38) | 81 (2) | 160 (3) | 771 (9) | 689 (9) | 824 (11) | 295 (12) | 378 (12) | 375 (24) |
| Maternal confidence | 5876 (38) | 82 (2) | 162 (4) | 766 (9) | 686 (9) | 822 (11) | 297 (13) | 373 (12) | 370 (24) |
| Maternal bonding | 5955 (39) | 122 (2) | 239 (5) | 589 (7) | 731 (10) | 865 (11) | 308 (13) | 393 (12) | 390 (26) |

Analysis

In an attempt to address biases in incomplete participation, missing data for all confounders in the models were imputed using multiple imputation by chained equations (MICE) (White et al., 2011). Predictor and outcome variables were not imputed. The software used for imputation was SPSS version 24. SPSS uses an MCMC (Markov chain Monte Carlo) algorithm known as fully conditional specification or chained equations imputation.

The first stage of imputation is to create multiple copies of the dataset, with the missing values replaced by imputed values. These are sampled from their predictive distribution based on the available observed data (thus using a bayesian approach) (Sterne et al., 2009). For continuous variables, linear regression was used, and for categorical variables logistic regression was used. Five data sets were imputed, as deemed adequate by previous research (Schafer, 1999).

The missing-data mechanism, in line with standard approaches to multiple imputation (Rubin, 1976; Spratt et al., 2010), was plausibly assumed to be Missing at Random (MAR) as opposed to missing completely at random (MCAR) or missing not at random (MNAR). This means the variables included in the imputation model have missing data which is conditional on another variable/s.

Complete cases analyses were also conducted for comparison with similar findings (data not shown). However, as analyses of complete cases suffer more from chance variation; under the missing at random assumption, it is generally accepted that multiple imputation should correct biases that may arise in complete cases analyses (Sterne et al., 2009).

ADDITIONAL RESULTS

Prevalence of child emotional health symptoms in the study population are presented in Supplementary Table 2 and 3.

**Supplementary Table 2**. Prevalence of child emotional health symptoms in the study population

| **Age** | **Measure** | ***N* (%)** |
| --- | --- | --- |
| 8 | Harter’s Self Perception Profile for Children Low scholastic competence | 998 (23.9) (score range= 6–24; ≤14 = low score) |
| 8 | Harter’s Self Perception Profile for Children Low global self-worth | 807 (20.3) (score range= 6–24; ≤16 = low score) |
| 10 | Moods and Feelings Questionnaire | 327 (4.4) (>11 indicates high levels of depressive symptoms) |
| 13 | Moods and Feelings Questionnaire | 551 (9.2) (>11 indicates high levels of depressive symptoms) |

**Supplementary Table 3.** Prevalence in the Study Population of Symptom-Based Outcome Variables (and a description of how they were derived from the DAWBA)

| **Age** | **Outcome Variables**  **Derived From**  **DAWBA** | **Prevalence in**  **ALSPAC Study**  **Population,**  *N* (%) | **Derivation of Dichotomous Outcome Variables From List of Symptoms in DAWBA and Examples of Items in DAWBA Relating to Each Outcome** |
| --- | --- | --- | --- |
| 7 | Separation anxiety | 542 (7.2) | Any separation anxiety symptom(s) “a lot more than others” compared with “no more than others” or “a little more than others,” for example, has he/she worried about sleeping alone? |
| 10 |  | 482 (6.6) |  |
| 13 |  | 259 (4) |  |
| 7 | Social fears | 438 (5.5) | Any social fears “a lot” compared with “none,” “a little,” or “hasn’t done this in the last month,” for example, has he/she been afraid of meeting new people? |
| 10 |  | 479 (6.2) |  |
| 13 |  | 581 (8.2) |  |
| 7 | Generalized anxiety | 687 (8.5) | Any of the worries “often” compared with “sometimes” or “not at all,” for example, does he/she worry a lot about schoolwork, homework, or tests/examinations? |
| 10 |  | 671 (20.3) |  |
| 13 |  | 510 (18) |  |
| 7 | Low mood/ depression | 955 (11.9) | Any mood symptoms compared with none, for example, did he/she think about death a lot? |

Supplementary results tables

Emotional health

**Supplementary Table 4.** Univariable and multivariable random effects hierarchical model for ‘Any’ pet ownership and Social Anxiety at age 7, 10, 13.

|  |  | **Univariable** |  |  | **Multivariable (adjusted)** |  |
| --- | --- | --- | --- | --- | --- | --- |
|  | Estimate | CI | *P* | Estimate | CI | *P* |
| **Social anxiety** |  |  | 0.004 |  |  | 0.621 |
| (intercept) | -2.91 | 0.07-3.06 |  | -4.93 | -7.11—2.76 |  |
| No | ref |  |  | ref |  |  |
| Yes | 0.24 | 0.08-0.41 |  | 0.09 | -0.29-0.49 |  |

Analyses adjusted for: sex, maternal depression measured at child age 8 and 11 years, maternal anxiety measured at child age 6 and 11 years, overcrowding (child age 7, 8 and 10 years), house type (child age 7 and 10 years), highest parental social class, maternal education, maternal age at delivery, financial difficulties, home ownership status, and car ownership, developmental delay measured at child age 30 months, older children living with child, stressful life events at child age 3, 9 and 11 years and maternal bonding measured at child age 3 years

**Supplementary Table 5.** Emotional health results showing univariable and multivariable associations between pet ownership at ages 7, 10 and 13 years and depression DAWBA (Development and Wellbeing Assessment) at ages 7, 10 and 13 years.

|  | **Emotional health outcome** |  | **Univariable** | | **Multivariable (adjusted)** | |
| --- | --- | --- | --- | --- | --- | --- |
| **Age** | **Depression (7 – DAWBA any mood symptoms; 10 & 13 – Moods and Feelings Questionnaire high score >11)** | *N* | OR (95% *CI*) | *p* | OR (95% *CI*) | *p* |
|  | Has any Pet |  |  |  |  |  |
| **7** |  | 7194 | 1.07 (0.91, 1.26) | 0.391 | 1.08 (0.91, 1.27) | 0.384 |
| **10** |  | 6054 | 0.96 (0.72, 1.18) | 0.789 | 0.94 (0.70, 1.26) | 0.666 |
| **13** |  | 2332 | 1.30 (0.92, 1.84) | 0.126 | 1.03 (0.72, 1.48) | 0.856 |
|  | Has Dog |  |  |  |  |  |
| **7** |  | 7192 | 1.07 (0.90, 1.27) | 0.406 | 1.09 (0.91, 1.29) | 0.349 |
| **10** |  | 6053 | 0.98 (0.73, 1.32) | 0.932 | 0.96 (0.71, 1.29) | 0.776 |
| **13** |  | 2337 | 1.12 (0.83, 1.51) | 0.447 | 0.94 (0.69, 1.29) | 0.695 |
|  | Has Cat |  |  |  |  |  |
| **7** |  | 7191 | 1.12 (0.96, 1.31) | 0.129 | 1.05 (0.90, 1.23) | 0.504 |
| **10** |  | 6053 | 1.02 (0.78, 1.34) | 0.868 | 0.93 (0.71, 1.23) | 0.617 |
| **13** |  | 2338 | 1.16 (0.87, 1.55) | 0.302 | 0.98 (0.73, 1.32) | 0.895 |
|  | Has other/miscellaneous pets |  |  |  |  |  |
| **7** |  | 7192 | 1.03 (0.89, 1.18) | 0.673 | 1.07 (0.92, 1.24) | 0.384 |
| **10** |  | 6053 | 1.84 (0.84, 1.39) | 0.532 | 1.10 (0.86, 1.43) | 0.449 |
| **13** |  | 2336 | 1.08 (0.82, 1.42) | 0.572 | 0.97 (0.73, 1.29) | 0.828 |

Analyses of depression measures adjusted for: sex, maternal depression measured at child age 8 and 11 years, maternal anxiety measured at child age 6 and 11 years, overcrowding (child age 7, 8 and 10 years), house type (child age 7 and 10 years), highest parental social class (antenatal period), maternal education (antenatal period), maternal age at delivery (antenatal period), financial difficulties (antenatal period), home ownership status (antenatal period), and car ownership (antenatal period), developmental delay measured at child age 30 months, older children living with child, stressful life events at child age 3, 9 and 11 years and maternal bonding measured at child age 3 years. Results did not differ when accounting for parental marital status, child temperament and dog walking (Number of times in typical week respondent walked or jogged with household dog(s)), therefore these variables were discarded from the final models.

**Supplementary Table 6.** Univariable and multivariable associations between pet ownership history (always, sometimes, and never owned pets up to 13 years) and the likelihood of anxiety and depression at 13 years.

|  |  |  | Univariable | | Multivariable (adjusted) | |
| --- | --- | --- | --- | --- | --- | --- |
| **Pet Ownership history** |  | *N* | OR (95% CI) | *p* | OR (95% CI) | *p* |
|  | **Separation Anxiety** | 1783 |  |  |  |  |
| Never |  |  | 1 |  | 1 |  |
| Sometimes |  |  | 1.55 (0.68, 3.54) | 0.298 | 0.82 (0.52, 1.30 | 0.399 |
| Always |  |  | 1.61 (0.74, 3.49) | 0.234 | 0.90 (0.59, 1.38) | 0.634 |
|  | **Social Anxiety** | 1936 |  |  |  |  |
| Never |  |  | 1 |  | 1 |  |
| Sometimes |  |  | 0.93 (0.59, 1.46) | 0.761 | 0.82 (0,52, 1.30) | 0.399 |
| Always |  |  | 1.01 (0.67, 1.52) | 0.981 | 0.90 (0.59, 1.38) | 0.634 |
|  | **Generalized Anxiety Disorder** | 849 |  |  |  |  |
| Never |  |  | 1 |  | 1 |  |
| Sometimes |  |  | 1.31 (0.83, 2.07) | 0.250 | 1.17 (0.72, 1.89) | 0.529 |
| Always |  |  | 0.99 (0.63, 1.55) | 0.963 | 0.89 (0.55, 1.42) | 0.610 |
|  | **Depression** | 1706 |  |  |  |  |
| Never |  |  | 1 |  | 1 |  |
| Sometimes |  |  | 0.93 (0.59, 1.46) | 0.765 | 0.75 (0.47, 1.19) | 0.220 |
| Always |  |  | 1.09 (0.73, 1.63) | 0.677 | 0.84 (0.55, 1.29) | 0.425 |

Analyses adjusted for: sex, maternal depression measured at child age 11 years, maternal anxiety measured at 11 years, overcrowding (10 years), house type (10 years), highest parental social class, maternal education, maternal age at delivery, financial difficulties, home ownership status, and car ownership, developmental delay measured at child age 30 months, older children living with child, stressful life events at 11 years and maternal bonding measured at child age 3 years.

**Supplementary Table 7**. Associations between Pet Interaction at age 6 and the likelihood of separation anxiety, social anxiety, generalized anxiety disorder and depression at age 7

|  | Univariable | | | Multivariable (adjusted) | | |  |
| --- | --- | --- | --- | --- | --- | --- | --- |
|  | *N* | OR (95% *CI*) | *p* | *N* | OR (95% *CI*) | *p* | |
|  |  |  |  |  |  |  | |
| **Separation Anxiety** | 4969 |  |  | 4442 |  |  | |
| Often looks after pets |  | 1 |  |  | 1 |  | |
| Occasionally |  | 1.03 (0.73, 1.45) | 0.885 |  | 1.03 (0.71, 1.51) | 0.862 | |
| Not at all |  | 1.16 (0.83, 1.60) | 0.384 |  | 1.17 (0.81, 1.68) | 0.401 | |
| **Social Anxiety** | 5370 |  |  | 4442 |  |  | |
| Often looks after pets |  | 1 |  |  | 1 |  | |
| Occasionally |  | 0.80 (0.57, 1.13) | 0.202 |  | 0.82 (0.57, 1.19) | 0.309 | |
| Not at all |  | 0.79 (0.58, 1.09) | 0.164 |  | 0.75 (0.53, 1.08) | 0.120 | |
| **Generalized Anxiety** | 5404 |  |  | 4819 |  |  | |
| Often looks after pets |  | 1 |  |  | 1 |  | |
| Occasionally |  | 1.06 (0.79, 1.41) | 0.709 |  | 1.15 (0.83, 1.59) | 0.396 | |
| Not at all |  | 0.97 (0.74, 1.29) | 0.868 |  | 1.04 (0.76, 1.42) | 0.804 | |
| **Depression** | 5366 |  |  | 4785 |  |  | |
| Often looks after pets |  | 1 |  |  | 1 |  | |
| Occasionally |  | 0.91 (0.71, 1,16) | 0.442 |  | 0.91 (0.69, 1.18) | 0.480 | |
| Not at all |  | 0.93 (0.74, 1.17) | 0.555 |  | 0.95 (0.73, 1.22) | 0.657 | |

Analyses adjusted for: sex, maternal depression measured at child age 8 years, maternal anxiety measured at child age 6 years, overcrowding (child age 7 years), house type (child age 7 years), highest parental social class, maternal education, maternal age at delivery, financial difficulties, home ownership status, and car ownership, developmental delay measured at child age 30 months, older children living with child, stressful life events at child age 3 years, and maternal bonding measured at child age 3 years

Behavioural health

Prevalence of child behavioural difficulties in the study population are presented in Supplementary data Table 7.

**Supplementary Table 8**. Prevalence of child behavioural health difficulties in the study population

|  | ***N* (%)** | |
| --- | --- | --- |
| **Outcome** | **Age 3 (RRS)** | **Age 11 (SDQ)** |
| Emotional Difficulties | 4593 (46) | 913 (12) |
| Hyperactivity | 4775 (48) | 882 (12) |
| Conduct Difficulties | 4969 (57) | 1177 (16) |
| Prosocial Difficulties | 3976 (44) | 541 (7) |
| Peer Problems | -- | 1158 (16) |
| Total Difficulties | 3932 (39) | 712 (10) |

Pet Ownership History

**Supplementary Table 9.** Univariable and multivariable associations (binary logistic regression) between pet ownership history (always, versus sometimes owned pets up to 11 years as reference) and behavioural outcomes at 11 years

|  | |  |  | | Univariable | | | Multivariable (adjusted) | | | | |
| --- | --- | --- | --- | --- | --- | --- | --- | --- | --- | --- | --- | --- |
| **Pet ownership history** | |  | *N* | | OR (95% CI) | | *p* | OR (95% CI) | | *p* | | |
|  | | **Emotional difficulties** | 2002 | |  | |  |  | |  | | |
| Sometimes | |  |  | | 1 | |  | 1 | |  | | |
| Always | |  |  | | 1.15 (0.85. 1.45) | | 0.436 | 1.11 (0.82, 1.50) | | 0.497 | | |
|  | | **Conduct disorder** | 2002 | |  | |  |  | |  | | |
| Sometimes | |  |  | | 1 | |  | 1 | |  | | |
| Always | |  |  | | 1.37 (1.04, 1.81) | | 0.024* | 1.26 (0.94, 1.69) | | 0.120 | | |
|  | | **Hyperactivity** | 2000 | |  | |  |  | |  | | |
| Sometimes | |  |  | | 1 | |  | 1 | |  | | |
| Always | |  |  | | 1.06 (0.76, 1.48) | | 0.736 | 1.05 (0.73, 1.50) | | 0.799 | | |
|  | **Peer Problems** | | 2002 |  | |  | | |  | |  |  |
| Sometimes |  | |  | 1 | |  | | | 1 | |  |  |
| Always |  | |  | 0.96 (0.76, 1.24) | | 0.798 | | | 0.98 (0.75, 1.27) | | 0.851 |  |
|  | | **Prosocial** | 2003 | |  | |  |  | |  | |  |
| Sometimes | |  |  | | 1 | |  | 1 | |  | |  |
| Always | |  |  | | 0.79 (0.54, 1.17) | | 0.247 | 0.88 (0.58, 1.32) | | 0.526 | |  |
|  | | **Total behaviour difficulties** | 2002 | |  | |  |  | |  | |  |
| Sometimes | |  |  | | 1 | |  | 1 | |  | |  |
| Always | |  |  | | 1.07 (0.76, 1.52) | | 0.695 | 0.94 (0.65, 1.37) | | 0.754 | |  |

Analyses adjusted for: sex, birthweight, maternal depression measured at child age 2 and 11 years, maternal anxiety measured at child age 2 and 11 years, overcrowding (child age 2 and 10 years), highest parental social class, maternal education, maternal age at delivery, family income, housing defects, financial difficulties, home ownership status, car ownership, developmental delay measured at child age 30 months, child temperament at 2 years, older children living with child, stressful life events at child age 2 and 11 years and maternal bonding measured at child age 3 years.

Supplementary Table 10. Univariable and multivariable associations between pet ownership at ages 8, 10 and 11, and attention and impulsivity(For working memory please see..

|  |  |  | **Univariable** | | **Multivariable (adjusted)** | |
| --- | --- | --- | --- | --- | --- | --- |
| **Age** |  | N | b (95% *CI*) | *p* | b (95% *CI*) | *p* |
|  | **Impulsivity** | | | | | |
| **10** | **Stop-signal task 150ms delay (number of correct trials)** |  |  |  |  |  |
|  | Has any Pet | 5729 | 0.07 (-0.12, 0.25) | 0.464 | 0.45 (-0.15, 0.24) | 0.655 |
|  | Has Dog | 5729 | 0.07 (-0.12, 0.25) | 0.464 | -0.04 (-0.23, 0.16) | 0.727 |
|  | Has Cat | 5728 | 0.02 (-0.15, 0.19) | 0.850 | 0.02 (-0.16, 0.20) | 0.810 |
|  | Has other/miscellaneous pets | 5728 | 0.18 (0.02, 0.34) | 0.028* | 0.16 (-0.01, 0.33) | 0.057 |
| **10** | **Stop-signal task 250ms delay (number of correct trials)** |  |  |  |  |  |
|  | Has any Pet | 5729 | 0.08 (-0.08, 0.24) | 0.344 | 0.04 (-0.13, 0.20) | 0.659 |
|  | Has Dog | 5728 | -0.07 (-0.23, 0.09) | 0.382 | -0.10 (-0.27, 0.07) | 0.245 |
|  | Has Cat | 5728 | 0.02 (-0.12, 0.17) | 0.747 | 0.02 (-0.14, 0.17) | 0.847 |
|  | Has other/miscellaneous pets | 5728 | 0.11 (-0.03, 0.25) | 0.132 | 0.07 (-0.07, 0.22) | 0.321 |
|  | **Working Memory** | | | | | |
|  | **Digit span task (correctly recalled sets weighted by number of screens within each set)** |  |  |  |  |  |
|  | Has any Pet |  |  |  |  |  |
| **8** |  | 5749 | -0.51 (-16.06, 15.05) | 0.989 | -2.56 (-18.26, 13.14) | 0.749 |
| **10** |  | 5760 | 11.21 (-2.31, 24.72) | 0.104 | 3.77 (-8.37, 15.91) | 0.543 |
|  | Has Dog |  |  |  |  |  |
| **8** |  | 5749 | -1.60 (-18.08, 14.88) | 0.849 | -3.55 (-20.31, 13.20) | 0.678 |
| **10** |  | 5759 | 6.79 (-6.89, 20.50) | 0.330 | 3.62 (-8.92, 16.16) | 0.571 |
|  | Has Cat |  |  |  |  |  |
| **8** |  | 5749 | -6.26 (-21.01, 8.50) | 0.406 | -8.14 (-22.93, 6.65) | 0.281 |
| **10** |  | 5759 | -0.37 (-13.02, 12.28) | 0.955 | -3.82 (-15.19, 7.55) | 0.510 |
|  | Has other/miscellaneous pets |  |  |  |  |  |
| **8** |  | 5749 | 0.98 (-12.71, 14.68) | 0.888 | -0.77 (-14.60, 13.05) | 0.912 |
| **10** |  | 5759 | 3.19 (-8.61, 14.99) | 0.596 | -1.01 (-11.71, 9.51) | 0.839 |

*P<0.05

Analyses were adjusted for: sex, maternal depression at ages 8 and 11 years, maternal anxiety at ages 6 and 11 years, overcrowding at 8 and 10 years, house type at ages 7 and 10 years, highest parental social class (antenatal period), maternal education (antenatal period), maternal age at delivery (antenatal period), home ownership status (antenatal period), family income (antenatal period) and car ownership (antenatal period), birthweight, developmental delay measured at child age 30 months, child temperament at 2 years, older children living in the house (antenatal period), stressful life events at almost 4 years, 9 and 11 years old, and mother-child bonding at child age 3 years.

Results did not differ when accounting for parental marital status and financial difficulties therefore these variables were discarded from the final models.

**Supplementary Table 11.** Univariable and multivariable associations between pet ownership history (always versus, sometimes owned pets up to 11 years as reference) and cognitive outcomes (attention) at 11 years.

|  |  | Univariable | | Multivariable (adjusted) | |
| --- | --- | --- | --- | --- | --- |
| **Pet ownership history** | N | B (95% *CI*) | *p* | B (95% *CI*) | *p* |
| Sky Search | 1854 | -0.09 (-0.19, 0.01) | 0.054 | -0.08 (-0.18, 0.02) | 0.112 |
| Attentional switching (Dual task) | 1742 | 0.25 (-0.07, 0.57) | 0.131 | 0.28 (-0.05, 0.60) | 0.098 |
| Same worlds task | 1803 | 0.15 (-0.01, 0.31) | 0.071 | 0.07 (-0.09, 0.24) | 0.364 |
| Opposite worlds task | 1802 | 0.20 (-0.02, 0.43) | 0.077 | 0.11 (-0.12, 0.34) | 0.342 |

Analyses were adjusted for: sex, maternal depression at 11 years, maternal anxiety at 11 years, overcrowding at 10 years, house type at 10 years, highest parental social, maternal education, maternal age at delivery, home ownership status, family income and car ownership, birthweight, developmental delay, child temperament, older children living in the house, stressful life events at 11 years old, and mother-child bonding at child age 3 years.

Educational

**Supplementary Table 12.** Univariable and multivariable associations between pet ownership history (always versus sometimes owned pets up to 15 years as reference) and GCSE attainment.

|  |  | Univariable | | Multivariable (adjusted) | |
| --- | --- | --- | --- | --- | --- |
| **Pet ownership history** | N | OR (95% *CI*) | *p* | OR (95% *CI*) | *p* |
| English | 1714 | 0.62 (0.51, 0.76) | <0.001 | 0.79 (0.63, 0.99) | 0.037 |
| Maths | 1654 | 0.51 (0.41, 0.62) | <0.001 | 0.67 (0.53, 0.85) | 0.001 |
| Biological Sciences | 433 | 0.71 (0.48, 1.05) | 0.089 | 0.87 (0.55, 1.36) | 0.533 |
| Chemistry | 424 | 0.73 (0.49, 1.08) | 0.113 | 0.79 (0.51, 1.24) | 0.315 |
| Physics | 423 | 0.95 (0.64, 1.40) | 0.796 | 1.09 (0.69, 1.71) | 0.694 |
| Achieved 5 GCSEs A*-C | 2021 | 0.64 (0.50, 0.82) | <0.001 | 0.80 (0.61, 1.05) | 0.109 |

Analyses were adjusted for: sex, maternal depression at 11 years, maternal anxiety at 11 years, overcrowding at 10 years, house type at 10 years, highest parental social class, maternal education, maternal age at delivery, home ownership status, family income and car ownership, school identifier, school type, birthweight, developmental delay, child temperament, older children living in the house, stressful life events at 11 years old, and mother-child bonding at age 3.

References

Angold, A., Costello, E. J., Messer, S. C., Pickles, A., Winder, F., & Silver, D. (1995). The development of a short questionnaire for use in epidemiological studies of depression in children and adolescents. *International Journal of Methods in Psychiatric Research, 5*, 237-249.

Birtchnell, J., Evans, C., & Kennard, J. (1988, Sep). The total score of the Crown-Crisp Experiential Index: a useful and valid measure of psychoneurotic pathology. *British Journal of Medical Psychology, 61 ( Pt 3)*, 255-266.

Carey, W. B., & McDevitt, S. C. (1978, May). Revision of the Infant Temperament Questionnaire. *Pediatrics, 61*(5), 735-739.

Cox, J. L., Holden, J. M., & Sagovsky, R. (1987, 06//). Detection of Postnatal Depression: Development of the 10-item Edinburgh Postnatal Depression Scale. *British Journal of Psychiatry, 150*, 782. <https://liverpool.idm.oclc.org/login?url=http://search.ebscohost.com/login.aspx?direct=true&db=edb&AN=25001374&site=eds-live&scope=site>

Fenson, L., Marchman, V. A., Thal, D. J., & Dale, P. S. (1991). *MacArthur-Bates communicative development inventories: User’s guide and technical manual.* . San Diego State University.

Frankenburg, W. K., & Dodds, J. B. (1967, 08 / 01 /). The Denver Developmental Screening Test [Article]. *The Journal of Pediatrics, 71*(2), 181-191. <https://liverpool.idm.oclc.org/login?url=http://search.ebscohost.com/login.aspx?direct=true&db=edselc&AN=edselc.2-52.0-0014118255&site=eds-live&scope=site>

Goodman, R., Ford, T., Richards, H., Gatward, R., & Meltzer, H. (2000, Jul). The Development and Well-Being Assessment: description and initial validation of an integrated assessment of child and adolescent psychopathology. *J Child Psychol Psychiatry, 41*(5), 645-655.

Harter, S. (1985). *Self-Perception Profile for Children.* University of Denver.

Joinson, C., Sullivan, S., von Gontard, A., & Heron, J. (2016). Stressful Events in Early Childhood and Developmental Trajectories of Bedwetting at School Age. *Journal of Pediatric Psychology, 41*(9), 1002-1010. <https://doi.org/10.1093/jpepsy/jsw025>

Manly, T., Robertson, I. H., Anderson, V., & Nimmo-Smith, I. (1998). *The Test of Everyday Attention for Children (TEAch)*. Thames Valley Test Company.

Messer, S. C., Angold, A., Costello, E. J., & Loeber, R. (1995). Development of a Short Questionnaire for Use in Epidemiological Studies of Depression in Children and Adolescents: Factor Composition and Structure Across Development. *International Journal of Methods in Psychiatric Research, 5*, 251-262.

OPCS. (1991). OPCS Standard occupational classification Her Majesty's Stationary Office, London.

Reynell, J., & Curwen, M. P. (1977). *Manual for the Reynell developmental language scales (revised)*. NFER.

Rubin, D. B. (1976). Inference and missing data. *Biometrika, 63*(3), 581-592.

Rutter, M., Tizard, J., & Whitmore, K. (1970). *Education, Health and Behaviour*. Longman.

Schafer, J. L. (1999). Multiple imputation: a primer. *Statistical methods in medical research, 8*(1), 3-15.

Spratt, M., Carpenter, J., Sterne, J. A. C., Carlin, J. B., Heron, J., Henderson, J., & Tilling, K. (2010). Strategies for Multiple Imputation in Longitudinal Studies. *American Journal of Epidemiology, 172*(4), 478-487. <https://doi.org/10.1093/aje/kwq137>

Sterne, J., White, I., Carlin, J., Spratt, M., Royston, P., Kenward, M., Wood, A., & Carpenter, J. (2009). Multiple imputation for missing data in epidemiological and clinical research: potential and pitfalls. *BMJ, 338*, b2393. <https://doi.org/10.1136/bmj.b2393>

White, I. R., Royston, P., & Wood, A. M. (2011). Multiple imputation using chained equations: Issues and guidance for practice. *STATISTICS IN MEDICINE, 30*(4), p377-p399.
